# Supplementary material for: The bidirectional association between premenstrual disorders and perinatal depression: A nationwide register-based study from Sweden
Source: PLoS Med. 2024 Mar 28;21(3):e1004363. doi: 10.1371/journal.pmed.1004363 (PMC10978009; doi:10.1371/journal.pmed.1004363)
Supplement: S1 Checklist — (DOCX) [file pmed.1004363.s001.docx]

S1 Checklist: STROBE Statement—checklist of items that should be included in reports of observational studies

|  | Item No | Recommendation | Section and Paragraph |
| --- | --- | --- | --- |
| **Title and abstract** | 1 | (a) Indicate the study’s design with a commonly used term in the title or the abstract | Title and Abstract |
|  |  | (b) Provide in the abstract an informative and balanced summary of what was done and what was found | Abstract para. 2-3 |
| Introduction | | | |
| Background/rationale | 2 | Explain the scientific background and rationale for the investigation being reported | Introduction para. 1-2 |
| Objectives | 3 | State specific objectives, including any prespecified hypotheses | Introduction para. 3 |
| Methods | | | |
| Study design | 4 | Present key elements of study design early in the paper | Study design in Methods |
| Setting | 5 | Describe the setting, locations, and relevant dates, including periods of recruitment, exposure, follow-up, and data collection | Data sources in Methods |
| Participants | 6 | (a) Cohort study—Give the eligibility criteria, and the sources and methods of selection of participants. Describe methods of follow-up  Case-control study—Give the eligibility criteria, and the sources and methods of case ascertainment and control selection. Give the rationale for the choice of cases and controls  Cross-sectional study—Give the eligibility criteria, and the sources and methods of selection of participants | Data sources and Study design in Methods |
|  |  | (b) Cohort study—For matched studies, give matching criteria and number of exposed and unexposed  Case-control study—For matched studies, give matching criteria and the number of controls per case | Study design in Methods |
| Variables | 7 | Clearly define all outcomes, exposures, predictors, potential confounders, and effect modifiers. Give diagnostic criteria, if applicable | Ascertainment of PND and Ascertainment of PMDs in Methods |
| Data sources/ measurement | 8* | For each variable of interest, give sources of data and details of methods of assessment (measurement). Describe comparability of assessment methods if there is more than one group | Data sources in Methods |
| Bias | 9 | Describe any efforts to address potential sources of bias | Adjustment, Sibling analysis and Additional analysis in Methods |
| Study size | 10 | Explain how the study size was arrived at | Data sources in Methods |
| Quantitative variables | 11 | Explain how quantitative variables were handled in the analyses. If applicable, describe which groupings were chosen and why | Statistical analysis in Methods |
| Statistical methods | 12 | (a) Describe all statistical methods, including those used to control for confounding | Statistical analysis in Methods |
|  |  | (b) Describe any methods used to examine subgroups and interactions | Additional analysis in Methods |
|  |  | (c) Explain how missing data were addressed | Covariates in Methods |
|  |  | (d) Cohort study—If applicable, explain how loss to follow-up was addressed  Case-control study—If applicable, explain how matching of cases and controls was addressed  Cross-sectional study—If applicable, describe analytical methods taking account of sampling strategy | Study design in Methods |
|  |  | (e) Describe any sensitivity analyses | Additional analysis in Methods |

Continued on next page

| Results | | | |
| --- | --- | --- | --- |
| Participants | 13* | (a) Report numbers of individuals at each stage of study—eg numbers potentially eligible, examined for eligibility, confirmed eligible, included in the study, completing follow-up, and analysed | Data sources in Methods |
|  |  | (b) Give reasons for non-participation at each stage | Data sources in Methods |
|  |  | (c) Consider use of a flow diagram | S1 Fig |
| Descriptive data | 14* | (a) Give characteristics of study participants (eg demographic, clinical, social) and information on exposures and potential confounders | Characteristics in Results |
|  |  | (b) Indicate number of participants with missing data for each variable of interest | Table 1 |
|  |  | (c) Cohort study—Summarise follow-up time (eg, average and total amount) | PND and subsequent risk of PMDs Section in Results |
| Outcome data | 15* | Cohort study—Report numbers of outcome events or summary measures over time | PND and subsequent risk of PMDs Section in Results & Table 4 |
|  |  | Case-control study—Report numbers in each exposure category, or summary measures of exposure | PMDs and subsequent risk of PND section in Results & Table 2 |
|  |  | Cross-sectional study—Report numbers of outcome events or summary measures |  |
| Main results | 16 | (a) Give unadjusted estimates and, if applicable, confounder-adjusted estimates and their precision (eg, 95% confidence interval). Make clear which confounders were adjusted for and why they were included | Table 2-6 |
|  |  | (b) Report category boundaries when continuous variables were categorized | Table 2, 4 |
|  |  | (c) If relevant, consider translating estimates of relative risk into absolute risk for a meaningful time period | Table 2-6 |
| Other analyses | 17 | Report other analyses done—eg analyses of subgroups and interactions, and sensitivity analyses | Additional analyses in Results |
| Discussion | | | |
| Key results | 18 | Summarise key results with reference to study objectives | Discussion para. 1 |
| Limitations | 19 | Discuss limitations of the study, taking into account sources of potential bias or imprecision. Discuss both direction and magnitude of any potential bias | Discussion para. 6 |
| Interpretation | 20 | Give a cautious overall interpretation of results considering objectives, limitations, multiplicity of analyses, results from similar studies, and other relevant evidence | Discussion para. 5 |
| Generalisability | 21 | Discuss the generalisability (external validity) of the study results | Discussion para. 6 |
| Other information | | | |
| Funding | 22 | Give the source of funding and the role of the funders for the present study and, if applicable, for the original study on which the present article is based | Included in the manuscript submission form as instructed by the journal |
