# Supplementary material for: The bidirectional association between premenstrual disorders and perinatal depression: A nationwide register-based study from Sweden
Source: PLoS Med. 2024 Mar 28;21(3):e1004363. doi: 10.1371/journal.pmed.1004363 (PMC10978009; doi:10.1371/journal.pmed.1004363)
Supplement: S1 Methods — (DOCX) [file pmed.1004363.s002.docx]

S1 Methods

Data origin and rational of the choice of covariates

Information on social demographics including maternal age, country of birth, cohabitation status, region of residence and educational level was obtained from the MBR (when possible) and the Longitudinal Integrated Database for Health Insurance and Labor Market Studies. Maternal smoking, body mass index (BMI), and parity are potential confounders for the association between PND and subsequent risk of PMDs.[1,2] The information on smoking three months before the pregnancy, BMI in early pregnancy, and parity was obtained from the MBR. PMDs are often comorbid with psychiatric disorders.[3] Previous psychiatric disorders, particularly depression, are risk factors for PND.[4] Therefore, we identified history of psychiatric disorders before pregnancy from the Patient Register from 1981 onward. Lastly, adverse pregnancy complications or delivery outcomes might confound or mediate the bidirectional association between PMDs and PND through mechanisms different from the abnormal response to hormonal changes. We therefore obtained information on hypertensive and diabetic diseases, preterm birth (gestational week <37 weeks), stillbirth, and low birth weight (birth weight<2500 grams) from the MBR. Congenital malformations of the offspring were retrieved from the MBR and the Patient Register, while neonatal death of the offspring (death within 28 days of birth) was identified from the MBR and the Cause of Death Register.

Reference

1. Yonkers, K.A. and M.K. Simoni, *Premenstrual disorders.* Am J Obstet Gynecol, 2018. **218**(1): p. 68-74.

2. Yang, K., J. Wu, and X. Chen, *Risk factors of perinatal depression in women: a systematic review and meta-analysis.* BMC Psychiatry, 2022. **22**(1): p. 63.

3. Pilver, C.E., D.J. Libby, and R.A. Hoff, *Premenstrual dysphoric disorder as a correlate of suicidal ideation, plans, and attempts among a nationally representative sample.* Soc Psychiatry Psychiatr Epidemiol, 2013. **48**(3): p. 437-46.

4. Beck, C.T., *Predictors of postpartum depression: an update.* Nurs Res, 2001. **50**(5): p. 275-85.
