# Supplementary material for: The bidirectional association between premenstrual disorders and perinatal depression: A nationwide register-based study from Sweden
Source: PLoS Med. 2024 Mar 28;21(3):e1004363. doi: 10.1371/journal.pmed.1004363 (PMC10978009; doi:10.1371/journal.pmed.1004363)
Supplement: S1 Fig — (DOCX) [file pmed.1004363.s003.docx]

S1 Fig. Flow chart

Excluded:

- 51,824 multiple births
- 34,790 pregnancies after diagnosis of perinatal depression
- 952 emigrated before the entry
- 344 pregnancies before age 15
- 39 pregnancies after age 52

1,041,419 individual women during 2001-2018, with 1,803,309 pregnancies

Cases: 85,949 incident cases of perinatal depression

Controls: 10 controls per cases individually matched on maternal age and calendar year; in total, 849,482 controls
