# Supplementary material for: The bidirectional association between premenstrual disorders and perinatal depression: A nationwide register-based study from Sweden
Source: PLoS Med. 2024 Mar 28;21(3):e1004363. doi: 10.1371/journal.pmed.1004363 (PMC10978009; doi:10.1371/journal.pmed.1004363)
Supplement: S1 Table — (DOCX) [file pmed.1004363.s004.docx]

S1 Table. International Classification of Diseases codes used to define the studied medical conditions

|  | ICD-8 | ICD-9 | ICD-10 |
| --- | --- | --- | --- |
| Calendar year | 1969-1986 | 1987-1996 | 1997-2014 |
| Malformations | - | - | Q00-Q99 |
| Hypertensive disease |  |  |  |
| Preeclampsia/eclampsia | 63703-63799 | 642E-642G | O14-O15 |
| Essential hypertension | 400-404 | 401-405, 642A-642C, 642H | O10-O11, I10-I15 |
| Diabetic diseases |  |  |  |
| Gestational diabetes |  | 648W | O244 |
| Diabetes |  | 250 | E10-E14 |
| Psychiatric disorders | 290-319 | 290-319 | F10-F90 |
| Depression |  |  | F32, F33 |

Abbreviations: CVD, cardiovascular diseases; ICD, *International Classification of Diseases*.
