# Supplementary material for: The bidirectional association between premenstrual disorders and perinatal depression: A nationwide register-based study from Sweden
Source: PLoS Med. 2024 Mar 28;21(3):e1004363. doi: 10.1371/journal.pmed.1004363 (PMC10978009; doi:10.1371/journal.pmed.1004363)
Supplement: S2 Table — (DOCX) [file pmed.1004363.s005.docx]

S2 Table. Association of premenstrual disorders (PMDs) with subsequent risk of perinatal depression (PND) adjusted for mediators: a nested case-control study.

|  | **Women without PND**  **N (%) of PMDs** | **Women with PND**  **N (%) of PMDs** | **Model 3 ^1^ OR (95% CIs)** |
| --- | --- | --- | --- |
| **Perinatal depression** | 5,199 (0.6) | 2,488 (2.9) | 3.88 (3.67,4.11) |
|  | | | |
| ***By time of diagnosis*** | | | |
| **Prenatal depression** | 3,052 (0.6) | 1,408 (3.0) | 3.42 (3.17,3.70) |
| *By time since pregnancy* | | | |
| 1^st^ trimester | 2,089 (0.8) | 870 (3.1) | 2.90 (2.63,3.20) |
| 2^nd^-3^rd^ trimester | 963 (0.5) | 538 (2.7) | 4.60 (4.04,5.23) |
|  |  |  |  |
| **Postnatal depression** | 2,147 (0.6) | 1,080 (2.9) | 4.49 (4.12,4.88) |
| *By time since delivery* | | | |
| ≤ 6 months | 1,044 (0.6) | 437 (2.4) | 3.73 (3.28,4.25) |
| 7-12 months | 1,103 (0.6) | 643 (3.3) | 5.40 (4.82,6.05) |

CIs, confidence intervals; N, number; OR, odds ratio; PMDs, premenstrual disorders; PND, perinatal depression.

^1^ Model 3 was adjusted for the matching variable (i.e., maternal age and calendar year), origin (Sweden or not), educational level (primary, high school, college and beyond), region of residence (south, middle, or north of Sweden), and cohabitation status (yes or no) at matching, parity (1, and ≥2), BMI during early pregnancy (categorized into <18.5, 18.5 to 24.9, 25 to 29.9, and ≥30 kg/m^2^), and smoking before pregnancy (no smoking, 1-9, and ≥10 cigarettes per day) and psychiatric disorder before pregnancy (yes or no). Estimates were obtained from logistic regression.
