# Supplementary material for: The bidirectional association between premenstrual disorders and perinatal depression: A nationwide register-based study from Sweden
Source: PLoS Med. 2024 Mar 28;21(3):e1004363. doi: 10.1371/journal.pmed.1004363 (PMC10978009; doi:10.1371/journal.pmed.1004363)
Supplement: S3 Table — (DOCX) [file pmed.1004363.s006.docx]

S3 Table. Stratified association of premenstrual disorders (PMDs) with subsequent perinatal depression (PND): a nested case control study.

|  | **Women without PND**  **N (%) of PMDs** | **Women with PND**  **N (%) of PMDs** | **OR (95% CIs) ^1^** |
| --- | --- | --- | --- |
| ***By parity*** | | |  |
| 1 | 2,206 (0.6) | 1,193 (2.7) | 4.13 (3.80,4.48) |
| ≥2 | 2,993 (0.6) | 1,295 (3.2) | 5.19 (4.81,5.61) |
| ***By calendar year at matching*** | | | |
| 2001-2010 | 1,065 (0.3) | 528 (1.4) | 4.89 (4.38,5.46) |
| 2011-2018 | 4,134 (0.9) | 1,960 (4.2) | 4.64 (4.37,4.93) |
| ***By maternal age at pregnancy, years*** | | | |
| 15-30 | 1,457 (0.4) | 783 (1.9) | 5.20 (4.73,5.72) |
| 31-52 | 3,742 (0.9) | 1,705 (3.9) | 4.50 (4.22,4.79) |

CIs, confidence intervals; N, number; OR, odds ratio; PMDs, premenstrual disorders; PND, perinatal depression.

^1^ Model was djusted for the matching variable (i.e., maternal age and calendar year), country of birth (Sweden or not), educational level (primary, high school, college and beyond), region of residence (south, middle, or north of Sweden), and cohabitation status (yes or no) at matching. Estimates were obtained from logistic regression.
