# Supplementary material for: The bidirectional association between premenstrual disorders and perinatal depression: A nationwide register-based study from Sweden
Source: PLoS Med. 2024 Mar 28;21(3):e1004363. doi: 10.1371/journal.pmed.1004363 (PMC10978009; doi:10.1371/journal.pmed.1004363)
Supplement: S4 Table — (DOCX) [file pmed.1004363.s007.docx]

S4 Table. Stratified association of perinatal depression (PND) with subsequent premenstrual disorders (PMDs): a matched cohort study.

|  | **Women without PND** | **Women with PND** | **HR (95% CIs) ^1^** |
| --- | --- | --- | --- |
|  | **N (IR) of PMDs** | **N (IR) of PMDs** |  |
| ***By parity*** | | |  |
| 1 | 9,759 (3.9) | 2,266 (8.0) | 1.79 (1.70,1.90) |
| ≥2 | 11,567 (3.8) | 1,961 (7.2) | 1.80 (1.70,1.90) |
| ***By calendar year at matching*** | | | |
| 2001-2010 | 13,304 (3.5) | 2,656 (6.8) | 1.83 (1.74,1.93) |
| 2011-2018 | 7,422 (4.8) | 1,532 (9.7) | 1.74 (1.63,1.86) |
| ***By maternal age at pregnancy, years*** | | | |
| 15-30 | 9,596 (3.6) | 1,936 (7.1) | 1.68 (1.58,1.78) |
| 31-52 | 11,251 (4.1) | 2,291 (8.2) | 1.91 (1.81,2.01) |

CIs, confidence intervals; HR, hazard ratio; IR, incidence rate, per 1000 person-years; N, number; PMDs, premenstrual disorders; PND, perinatal depression.

^1^ Model was adjusted for the matching variable (i.e., maternal age and calendar year), country of birth (Sweden or not), educational level (primary, high school, college and beyond), region of residence (south, middle, or north of Sweden), and cohabitation status (yes or no) at matching, parity (1, and ≥2), BMI during early pregnancy (categorized into <18.5, 18.5 to 24.9, 25 to 29.9, and ≥30 kg/m^2^), and smoking (no smoking, 1-9, and ≥10 cigarettes per day) and history of psychiatric disorders before pregnancy (yes or no). Estimates were obtained from Cox regression.
