# Supplementary material for: The bidirectional association between premenstrual disorders and perinatal depression: A nationwide register-based study from Sweden
Source: PLoS Med. 2024 Mar 28;21(3):e1004363. doi: 10.1371/journal.pmed.1004363 (PMC10978009; doi:10.1371/journal.pmed.1004363)
Supplement: S5 Table — (DOCX) [file pmed.1004363.s008.docx]

S5 Table. Bidirectional link between perinatal depression (PND) with premenstrual disorders (PMDs): sensitivity analysis.

| **PND subsequent to PMDs: a nested case control study** | | |
| --- | --- | --- |
| **Women without PND**  **N (%) of PMDs** | **Women with PND**  **N (%) of PMDs** | **OR (95% CIs) ^1^** |
| Restricting to women without pregnancy complications or adverse outcomes **^2^** | | |
| 4,332 (0.6) | 1,979 (2.9) | 4.84 (4.55,5.14) |
| Restricting to PMDs with two specialists-made diagnoses ≥28 days apart | | |
| 852 (0.1) | 503 (0.6) | 5.66 (5.04,6.36) |
| Restricting to PND identified with ICD code | | |
| 5,068 (0.6) | 2,348 (3.0) | 4.62 (4.38,4.87) |
| **PMDs subsequnt to PND: a matched cohort study** | | |
| **Women without PND N (IR)** | **Women with PND N (IR)** | **HR (95% CIs) ^3^** |
| Restricting to women without pregnancy complications or adverse outcomes **^2^** | | |
| 18,162 (3.9) | 3,442 (7.7) | 1.82 (1.74,1.90) |
| Restricting to PMDs with two specialists-made diagnoses ≥28 days apart | | |
| 2,961 (0.5) | 706 (1.3) | 2.18 (1.97,2.40) |
| Restricting to PND identified with ICD code | | |
| 19,428 (4.0) | 3,725 (7.9) | 1.79 (1.71,1.86) |

CIs, confidence intervals; HR, hazard ratio; IR, incidence rate, per 1000 person-years; N, number; OR, odds ratio; PMDs, premenstrual disorders; PND, perinatal depression.

^1^ Model was adjusted for the matching variable (i.e., maternal age and calendar year), country of birth (Sweden or not), educational level (primary, high school, college and beyond), region of residence (south, middle, or north of Sweden), and cohabitation status (yes or no) at matching.

^2^ Pregnancies with hypertensive and diabetic diseases, preterm birth, stillbirth and neonatal birth, low birth weight, diagnosis of major congenital malformations in the infant were excluded.

^3^ Model was adjusted for the matching variable (i.e., maternal age and calendar year), country of birth (Sweden or not), educational level (primary, high school, college and beyond), region of residence (south, middle, or north of Sweden), and cohabitation status (yes or no) at matching, parity (1, and ≥2), BMI during early pregnancy (categorized into <18.5, 18.5 to 24.9, 25 to 29.9, and ≥30 kg/m^2^), and smoking (no smoking, 1-9, and ≥10 cigarettes per day) and history of psychiatric disorders before pregnancy (yes or no). OR estimates were obtained from logistic regression and HR estimates were obtained from Cox regression.
