# Supplementary material for: The bidirectional association between premenstrual disorders and perinatal depression: A nationwide register-based study from Sweden
Source: PLoS Med. 2024 Mar 28;21(3):e1004363. doi: 10.1371/journal.pmed.1004363 (PMC10978009; doi:10.1371/journal.pmed.1004363)
Supplement: Attachment — Submitted filename: review comments.docx [file pmed.1004363.s010.docx]

Thank you for the opportunity to review this important piece of work on the bidirectional relationship between premenstrual disorders (PMDs) and perinatal depression (PND). I have provided detailed comments in the order of the main text sections. Analyses performed in this study is well presented however, the writing and logic of this paper warrants improvement. A major revision is my recommendation for the decision of publication of this paper in PLoS Medicine.

**Introduction**

1. Line 61, ‘Similarly, premenstrual disorders (PMDs) are characterized by … ’: please provide basic background knowledge about PMDs as ‘core PMDs’ is the term recently defined in the RCOG premenstrual disorder guideline.
2. Please add in a paragraph detailing the importance of researching the bidirectional relationship between PMD and PND. This echoes to what was illustrated in the Discussion section.
3. Line 69, ‘However, existing studies had retrospective design, …’: This is not for all previous studies – many of them adopted a prospective study design. The biggest advantage of this present study is that it uses the Swedish data registry which is a population-based sampling that to a large extend reduced the selection bias compared to any other previous studies.
4. Line 73, ‘no study has examined this hypothesis’: this statement is simply not true. Studies on the same topic have already been carried out in the 80s. See refs:

- Dennerstein L, Morse CA, Varnavides K. Premenstrual tension and depression — is there a relationship? *Journal of Psychosomatic Obstetrics & Gynecology*. 1988;8(1):45-52.
- Warner P, Bancroft J, Dixson A, Hampson M. The relationship between perimenstrual depressive mood and depressive illness. *Journal of Affective Disorders*. 1991;23(1):9-23.
- Haywood A, Slade P, King H. Is there evidence of an association between postnatal distress and premenstrual symptoms? *Journal of Affective Disorders*. 2007;99(1-3):241-245.

**Methods**

1. Ascertainment of PND

- Line 95, ‘in line with previous studies [ref 18], we identified PND from … using ICD-10 codes (F32, F33 and F59.0)’. This sentence is misleading as the paper being cited used a different set of ICD-10 codes to infer PND. Also, another paper the authors cited below ‘the diagnosis of major depression with used ICD codes has been validated in the Patient Register’ seems to be the right reference for this argument. After checking, their study only used ICD-10 codes F32-F33.9, which is also different the present study. Please correct. In addition, please justify why ICD-10 codes like F53, F34, F38 and F39 were not used to infer PND diagnoses. They are all frequently use for the ascertainment of PNDs [ref: Xu F, Austin M-P, Reilly N, et al. *Archives of Women’s Mental Health*. 2012;15(5):333.]
- Line 99, ‘although such definition of PND was not specifically validated, the diagnosis of major depression with used ICD codes has been validated in the Patient Register’: What does the result say?

2. Study design

- Line 130: Please explain what ‘the diagnosis/matching date’ means in detail. Why not simply state it as ‘matching date’? I assume no matter prenatal or postnatal depression, cases were matched on the diagnosis date by the gestational age for prenatal and postnatal day, respectively.

3. Statistical analysis

- Line 158, ‘Due to the design, the OR can be interpreted as HR…’: please elaborate on this using one or two sentences for the wider audience who is unfamiliar with epidemiology.
- Line 168, ‘stratified analysis by maternal age … ’: What was the relationship between maternal age (continuous variable) and PND? Which table shows this result?
- Line 170, ‘PND and subsequent risk of PMDs’: please explain why different study design were sued for the two research questions. Why not use matched cohort to explore PMDs🡪PND as well or use nested case-control for PND🡪PMDs?
- Line 175, ‘PND subtype’: add in brackets (i.e., those with and without a history of psychiatric disorders) if that is what the authors meant.

**Results**

1. PMDs and subsequent risk of PND

- Line 226, ‘was stronger among women without such history’: this result is very counter-intuitive and new findings as such (new finding no. 1) warrants a thorough discussion. Line 227, the association was also stronger among multiparous than primiparous women: same for this new finding (new finding no. 2) too.

2. PND and subsequent risk of PMDs

- PND🡪PMDs this association stronger in those without history of psychiatric disorders too: new finding worth discussing (new finding no. 3)
- Line 241, Table 4: how to explain also positive results among those without history of other psychiatric disorders (new finding no. 4)?

Discussion

1. Line 273, ‘to our knowledge, no study has … the association between prenatal depression and PMDs’: yes, but please expand a bit on why this is important/relevant.
2. Lines 281-286, ‘likely to take antidepressants, which may mitigate premenstrual symptoms’: I assume there are analyses of this paper that have accounted for this. E.g., to adjust for antidepressants use prior to the outcome.
