# Supplementary material for: The bidirectional association between premenstrual disorders and perinatal depression: A nationwide register-based study from Sweden
Source: PLoS Med. 2024 Mar 28;21(3):e1004363. doi: 10.1371/journal.pmed.1004363 (PMC10978009; doi:10.1371/journal.pmed.1004363)
Supplement: Attachment — Submitted filename: Response letter.docx [file pmed.1004363.s011.docx]

Manuscript ID: PMEDICINE-D-23-01426R2

Dear Dr. Dodd,

Thank you for the thorough review of our paper now entitled “**The bidirectional association between premenstrual disorders and perinatal depression: a nationwide register-based study from Sweden**” and for the opportunity to revise and resubmit an improved version for publication in *PLOS Medicine.*

We appreciate the positive remarks and insightful comments from the reviewers. Please find our point-by-point responses to those comments below. We have revised the manuscript in accordance with the comments from the editor and the reviewers, such as justifying the choice of study design, clarifying prospective data, and enriching the discussion on important aspects.

We believe that the quality of the manuscript has, as a result, been significantly improved. While we are open to make further revisions, we hope that you will find the present version of our manuscript satisfactory and suitable for publication in *PLOS Medicine*.

All authors have contributed to this revision and approved the final version of the manuscript.

Please do not hesitate to contact us if further modifications are needed.

Yours sincerely,

Qian Yang and Donghao Lu

On behalf of all co-authors

Institute of Environmental Medicine, Karolinska Institutet

Nobels väg 13, S-17 177 Stockholm, Sweden

*All page and line marks correspond to the line numbers of the ‘Revised manuscript_clean version’.

COMMENTS FROM THE EDITORS

GENERAL

1. Please respond to all editor and reviewer comments detailed below in full.

Authors’ response: Please find our point-by-point responses to those comments below.

2. Please ensure that the study is reported according to the STROBE guideline, and include the completed STROBE checklist as Supporting Information. Please add the following statement, or similar, to the Methods: "This study is reported as per the Strengthening the Reporting of Observational Studies in Epidemiology (STROBE) guideline (S1 Checklist)."

The STROBE guideline can be found here: <https://eur01.safelinks.protection.outlook.com/?url=http%3A%2F%2Fwww.equator-network.org%2Freporting-guidelines%2Fstrobe%2F&data=05%7C01%7Cqian.yang.1%40ki.se%7C025150da3b334e88df5308dbd63565d2%7Cbff7eef1cf4b4f32be3da1dda043c05d%7C0%7C0%7C638339297198185909%7CUnknown%7CTWFpbGZsb3d8eyJWIjoiMC4wLjAwMDAiLCJQIjoiV2luMzIiLCJBTiI6Ik1haWwiLCJXVCI6Mn0%3D%7C3000%7C%7C%7C&sdata=mwwkwK0BNwRU75NL5fkTbGaTD0iisQOQItlNFU%2FPNpM%3D&reserved=0>

When completing the checklist, please use section and paragraph numbers, rather than page numbers as these often change in the event of publication.

Authors’ response: We have completed the STOBE checklist as instructed and included the statement in “Methods” (page 10, line 208-209): “This study is reported as per the Strengthening the Reporting of Observational Studies in Epidemiology (STROBE) guideline (S1 Checklist).”

1. * The editorial team agree with the reviewer (please see below) regarding the need to better justify the rationale for the use of both a nested-case control study and a matched cohort study. Please include additional detail as relevant in the introduction, methods, and discussion sections of your manuscript.*

Authors’ response: Thank you for the suggestion. We have enriched our justification for the use of a nested case-control study and a matched cohort study design in the “Methods”.

“Introduction”, page 7, line 127-135: “Taken together, without prospective evidence, it remains unclear whether women with PMDs have an increased risk of developing PND when becoming pregnant or after giving birth, and vice versa. Using the Swedish nationwide registers with prospectively collected data, we aimed to investigate the bidirectional association between PMDs and PND. To study the bidirectional association in the same population effectively, we conducted a nested case-control study, a design inherently equivalent to a cohort study [1], to prospectively examine the risk of PND following PMDs. We then transitioned this design into a matched cohort study to assess the risk of PMDs after PND. We further employed sibling comparisons to account for shared genetic and familial environmental risk factors for both disorders.”

“Study design” in “Methods”, page 10, line 192-199: “To effectively examine the bidirectional association between PMDs and PND within the same study population, we conducted a nested case-control study, a design inherently equivalent to a cohort study [1], identifying all pregnancies, corresponding PNDs and previous history of any indications for PMDs to prospectively examine the risk of PND following PMDs. We then transitioned this design into a matched cohort with onward follow-up of index PNDs and control pregnancies, enabling us to efficiently assess the risk of PMDs after PND within the same study population.”

2. ** Please also take to care to ensure that you clearly and accurately differentiate between prospective and retrospective data collection and study design. Please see specific comments under ‘Introduction’ and ‘Discussion’.**

Authors’ response: We have revised the manuscript throughout to ensure clarity on prospective or retrospective data or design. Relevant sections include:

“Introduction”

Page 6, line 117-119: “However, existing studies relied on retrospectively collected on data premenstrual symptoms during or after pregnancy, which might be prone to recall bias and thereby biased results [2,3].”

“Discussion”

Page 16-17, line 345-356: “Previous studies have used data on history of PMDs retrospectively collected during or after pregnancy, which might be vulnerable to recall bias and systematic error [2,3]. Taking advantage of prospectively collected data in Swedish healthcare registers, the study is the first, to our knowledge, to demonstrate a positive association between PMDs and subsequent risk of PND. Moreover, previous studies exclusively focused on postnatal depression, mostly ascertained within 8 weeks postpartum [4,5]. With retrospectively collected data, one systematic review and one meta-analysis reported that women with postnatal depression were more likely to endorse a history of PMDs [4,5], which is in line with our results. The meta-analysis reported an OR of 2.20, 95% CI [1.81,2.68] for PMDs among women with postnatal depression (mostly assessed within 6 months after delivery) [4]. Correspondingly, our data show that women with PMDs have more than three times higher risk of PND within 6 months postpartum.”

Page 19, line 415-417: “The strength of the study lies in the large sample size with long and complete follow-up, the prospectively and independently collected data on PMDs and PND, and covariates that could confound the studied association.”

TITLE

3. include reference to Sweden in the title, we suggest, ‘The bidirectional association between premenstrual disorders and perinatal depression: a nationwide register-based study from Sweden’ or similar.

Authors’ response: Thank you for the suggestion. We have revised the title of the study as suggested.

COMPETING INTERESTS

All authors must declare their relevant competing interests per the PLOS policy, which can be seen here:

<https://eur01.safelinks.protection.outlook.com/?url=https%3A%2F%2Fjournals.plos.org%2Fplosmedicine%2Fs%2Fcompeting-interests&data=05%7C01%7Cqian.yang.1%40ki.se%7C025150da3b334e88df5308dbd63565d2%7Cbff7eef1cf4b4f32be3da1dda043c05d%7C0%7C0%7C638339297198185909%7CUnknown%7CTWFpbGZsb3d8eyJWIjoiMC4wLjAwMDAiLCJQIjoiV2luMzIiLCJBTiI6Ik1haWwiLCJXVCI6Mn0%3D%7C3000%7C%7C%7C&sdata=g3q9rKXqsgozvt5oW%2BTiYlXatregDCeVcLxseIVzd2g%3D&reserved=0>

For authors with ties to industry, please indicate whether any of the interests has a financial stake in the results of the current study.

Authors’ response: Thank you for the comment. The funding and declaration of completing interest were included in the submission form as required.

“Funding: The work was supported by the Chinese Scholarship Council (No. 201700260289 to Dr. Yang), the Swedish Research Council for Health, Working Life and Welfare (FORTE) (No. 2020-00971 and 2023-00399 to Dr. Lu), the Swedish Research Council (Vetenskapsrådet) (No. 2020-01003 to Dr. Lu), Karolinska Institutet Strategic Research Area in Epidemiology and Biostatistics (grant to Dr. Lu), and the Icelandic Research Fund (No. 218274-051 to Dr. Valdimarsdóttir). The funders had no role in the design of the study and collection, analysis, and interpretation of data and in writing the manuscript. The funders had no role in study design, data collection and analysis, preparation of the manuscript or decision to publish.

Competing interests: All authors declare no conflict of interest regarding this study.”

ABSTRACT

4. Please structure your abstract using the PLOS Medicine headings (Background, Methods and Findings, Conclusions).

Authors’ response: We have re-structured the abstract with PLOS Medicine headings.

5. Please combine the Methods and Findings sections into one section, “Methods and findings”.

Authors’ response: Thank you for the instruction. We have structured the abstract as instructed.

Abstract Background:

6. Provide the context of why the study is important, as in the current version. The final sentence should clearly state the study question.

Authors’ response: We have re-structured the background in the abstract as instructed.

Page 2, line 24-29: “Background: Premenstrual disorders (PMDs) and perinatal depression (PND) share symptomology and the timing of symptoms of both conditions coincide with natural hormonal fluctuations which may indicate a shared etiology. Yet, there is a notable absence of prospective data on the potential bidirectional association between these conditions, which is crucial for guiding clinical management. Using the Swedish nationwide registers with prospectively collected data, we aimed to investigate the bidirectional association between PMDs and PND.”

Abstract Methods and Findings:

7. Please ensure that all numbers presented in the abstract are present and identical to numbers presented in the main manuscript text.

Authors’ response: We have cross-checked the numbers presented in the abstract with results presented in the manuscript to make sure they are identical.

8. Please (briefly) justify the decision to use both methodological approaches in the study.

Authors’ response: We have added justification of two methodological approaches adopted in the study.

“Methods and findings”, page 2, line 30-34: “With 1,803,309 singleton pregnancies of 1,041,419 women recorded in the Swedish Medical Birth Register during 2001-2018, we conducted a nested case-control study to examine the risk of PND following PMDs, which is equivalent to a cohort study but with improved efficiency; and transitioned that design into a matched cohort study with onward follow-up to examine the risk of PMDs after PND (within the same study population).”

9. Please quantify the main results with 95% CIs and p values. When reporting p values please report as p<0.001 and where higher as p=0.002, for example. Please separate upper and lower CI bounds with commas as opposed to hyphens to prevent confusion with reporting of negative values. Suggest reporting statistical information as follows, ‘(OR 4.58; 95% CI [4.28,4.90];p</=) to improve accessibility and clarity for the reader.

Authors’ response: We have added p value and formatted the reported results as instructed.

Page 2-3, line 45-51: “In the nested case-control study, we identified 2,488 PMDs (2.9%) before pregnancy among women with PND and 5,199 (0.6%) among controls. PMDs were associated with a higher risk of subsequent PND (OR 4.76, 95% CI [4.52,5.01]; *p*<0.001). In the matched cohort with a mean follow-up of 7.40 years, we identified 4,227 newly diagnosed PMDs among women with PND (Incidence Rate (IR) 7.6/1000 person-years) and 21,326 among controls (IR 3.8). Compared to their matched controls, women with PND were at higher risk of subsequent PMDs (HR 1.81, 95% CI [1.74,1.88]; *p*<0.001).”

10. Please define ‘OR’, ‘IR’ and ‘HR’ at first use for the reader.

Authors’ response: We have defined all abbreviations at first use in the “Abstract”.

11. Please include any important dependent variables that are adjusted for in the analyses.

Authors’ response: We have described covariates adjusted in the model in the “Abstract” as suggested.

Page 2, line 37-43: “We 1) calculated Odds Ratio (OR) and 95% Confidence Interval (CI) of PMDs using conditional logistic regression in the nested case-control study. Demographic factors (country of birth, educational level, region of residency, and cohabitation status) were adjusted for. 2) calculated the Hazard Ratio (HR) and 95% CI of PMDs subsequent to PND using stratified Cox regression in the matched cohort study. Smoking, BMI, parity, and history of psychiatric disorders were further controlled for, in addition to demographic factors.”

12. Please include the actual amounts and/or absolute risk(s) of relevant outcomes, not just relative risks or correlation coefficients. (example for absolute risks: PMID: 28399126).

Authors’ response: We agree that the actual amounts and absolute risks are of readers’ interest and have presented them in the “Methods and findings”.

Please refer to the authors’ response to comment #10.

13. In the last sentence of the Abstract Methods and Findings section, please describe the main limitation(s) of the study's methodology.

Authors’ response: We have noted the main limitation of this study in the last sentence of the “Methods and findings” section in the “Abstract”.

Page 3, line 54-56:“The main limitation of this study was that our findings, based on clinical diagnoses recorded in registers, may not generalize well to women with mild PMDs or PND.”

Abstract Conclusions:

14. Please address the study implications without overreaching what can be concluded from the data; the phrase "In this study, we observed ..." may be useful.

Authors’ response: Thank you for the suggestion. We have revised as suggested.

Page 3, line 57-59: “Conclusions: In this study, we observed a bidirectional association between PMDs and PND. These findings suggest that a history of PMDs can inform PND susceptibility and vice versa, and lend support to the shared etiology between both disorders.”

15. Please interpret the study based on the results presented in the abstract, emphasizing what is new without overstating your conclusions.

Authors’ response: The point is well taken. Please refer to the authors’ response to comment #15.

16. Please avoid vague statements such as "these results have major implications for policy/clinical care". Mention only specific implications substantiated by the results.

Authors’ response: Thank you for the instruction. We believe the implications as written in the “Conclusions” in the “Abstract” were substantiated by the results of the study.

Please refer to the authors’ response to comment #15.

17. Please avoid assertions of primacy ("We report for the first time....")

Authors’ response: Assertions of primacy was not used in the “Abstract” and we have revised the manuscript throughout as suggested.

AUTHOR SUMMARY

1. At this stage, we ask that you include a short, non-technical Author Summary of your research to make findings accessible to a wide audience that includes both scientists and non-scientists. The authors summary should consist of 2-3 succinct bullet points under each of the following headings:

• Why Was This Study Done? Authors should reflect on what was known about the topic before the research was published and why the research was needed.

• What Did the Researchers Do and Find? Authors should briefly describe the study design that was used and the study’s major findings. Do include the headline numbers from the study, such as the sample size and key findings.

• What Do These Findings Mean? Authors should reflect on the new knowledge generated by the research and the implications for practice, research, policy, or public health. Authors should also consider how the interpretation of the study’s findings may be affected by the study limitations. In the final bullet point of ‘What Do These Findings Mean?’, please describe the main limitations of the study in non-technical language.

Author Summary should immediately follow the Abstract in your revised manuscript. This text is subject to editorial change and should be distinct from the scientific abstract. Please see our author guidelines for more information: <https://eur01.safelinks.protection.outlook.com/?url=https%3A%2F%2Fjournals.plos.org%2Fplosmedicine%2Fs%2Frevising-your-manuscript%23loc-author-summary&data=05%7C01%7Cqian.yang.1%40ki.se%7C025150da3b334e88df5308dbd63565d2%7Cbff7eef1cf4b4f32be3da1dda043c05d%7C0%7C0%7C638339297198185909%7CUnknown%7CTWFpbGZsb3d8eyJWIjoiMC4wLjAwMDAiLCJQIjoiV2luMzIiLCJBTiI6Ik1haWwiLCJXVCI6Mn0%3D%7C3000%7C%7C%7C&sdata=uD4b3ydxtKeY%2Bs0hVGDu8UnJbGT63ke%2FLE3dyfL0HYg%3D&reserved=0>

Authors’ response: We have added the “Author summary” section as instructed.

Page 4-5

**Author summary**

**Why was this study done?**

Perinatal depression (PND) and premenstrual disorders (PMDs) share symptomology (e.g., feeling depressed) and the timing of symptom onset of both conditions coincides with natural hormonal fluctuations.

Two recent systematic reviews showing that women with PND were more likely to report a history of PMDs. Yet, prospective data are lacking to navigate clinical management. Moreover, premenstrual symptoms can worsen after pregnancy due to an escalated abnormal response to hormonal changes in relation to pregnancy. However, it is largely unknown whether women with PND are at risk for subsequent PMDs.

**What did the researchers do and find?**

Among 1,803,309 singleton pregnancies of 1,041,419 women recorded in the Swedish Medical Birth Register during 2001-2018, we identified 84,949 incidence cases of PND. For each case, 10 controls that were free from PND at the time when the matched case was diagnosed were randomly selected for comparison. We then conducted 1) a nested case-control study to examine the association between PMDs and risk of PND; and 2) a matched cohort study to examine the association between PND and subsequent risk of PMDs. Pregnancies from full sisters of PND cases were also identified for sibling comparison which contrasts the risk within each set of full sisters discordant on PND.

Among women with PND, 2.9% had PMDs before pregnancy, in contrast to 0.6% among matched unaffected women. PMDs were associated with a higher risk of subsequent PND (OR 4.76, 95% CI [4.52,5.01]; p<0.001). In the matched cohort with a mean follow-up of 6.90 years, we identified 4,227 newly diagnosed PMDs among women with PND (Incidence Rate (IR) 7.6/1000 person-years), against 21,326 PMDs among controls (IR 3.8/1000 person-years). Compared to their matched unaffected women, women with PND were at higher risk of subsequent PMDs (HR 1.81, 95% CI [1.74,1.88]; p<0.001).

The bidirectional association between PMDs and PND was noted for both prenatal and postnatal depression, and was stronger among women without history of psychiatric disorders. Moreover, the sibling comparison showed somewhat attenuated, yet statistically significant, bidirectional association.

**What do these findings mean?**

The nationwide study using prospectively collected data, to our knowledge, is the first to illustrate a bidirectional association between PMDs and PND, including both prenatal and postnatal depression. These findings suggest that a history of PMDs can inform PND susceptibility and *vice versa*.

INTRODUCTION

1. In the discussion you make the distinction between using prospectively collected data as opposed to retrospectively collected data very clear (. Here is it less clear and could be misinterpreted. At first read I thought you were describing your study as prospective. Please revise for clarity Perhaps instead you could introduce the rationale for using a nested-case control and matched cohort design to address your questions in context of other existing study designs. What advantage does this offer above existing published studies? Please also see the methods section and reviewer comments (below) in reference to the same. You may decide that all this information is better placed there. We leave it to your discretion.

Authors’ response: Thank you for the suggestion. We have added the rationale for using a nested-case control and a matched cohort design for the aim of the study in the context of existing studies in the “Introduction” as below:

Page 6-7, line 117-135: “However, existing studies relied on retrospectively collected on data premenstrual symptoms during or after pregnancy, which might be prone to recall bias and thereby biased results [2,3]. Moreover, the community- or clinic-based sampling in previous studies may have introduced significant selection bias [6]. Premenstrual symptoms can worsen after pregnancy due to an escalated abnormal response to hormonal changes in relation to pregnancy [7]. It is thus plausible that women with PND are at risk for subsequent PMDs. However, few studies with a relatively small sample size have examined this hypothesis by comparing proportions without adjustment for confounders and generated inconsistent results [8-10].

Taken together, without prospective evidence, it remains unclear whether women with PMDs have an increased risk of developing PND when becoming pregnant or after giving birth, and vice versa. Using the Swedish nationwide registers with prospectively collected data, we aimed to investigate the bidirectional association between PMDs and PND. To study the bidirectional association in the same population effectively, we conducted a nested case-control study, a design inherently equivalent to a cohort study [1], to prospectively examine the risk of PND following PMDs. We then transitioned this design into a matched cohort study to assess the risk of PMDs after PND. We further employed sibling comparisons to account for shared genetic and familial environmental risk factors for both disorders.”

2. In revising your introduction, please ensure that you indicate whether your study is novel and how you determined that and as in the current version, please conclude the Introduction with a clear description of the study question or hypothesis.

Authors’ response: Please refer to the authors’ response to the comment above.

METHODS and RESULTS

1. As above please ensure that you add the following statement, or similar, to the Methods: "This study is reported as per the Strengthening the Reporting of Observational Studies in Epidemiology (STROBE) guideline (S1 Checklist)."

Authors’ response: We have added the statement as required. Please refer to the authors’ response to editors’ general comment #2.

2. As above, please clearly explain the rationale for implementing a nested-case control and a matched cohort design.

Authors’ response: We have explained the implantation of study design in the presented work in the “Study design” in “Methods” as suggested.

Please refer to the authors’ response to the editors’ general comment #2.

3. Line 81 – suggest, ‘Databases’ perhaps, instead, as a sub-heading.

Authors’ response: Thank you for the suggestion. We have renamed the section to “Data source and study population”.

4. Please see reviewer comments below regarding the use of DSM-5 and ICD-10 coding and the potential implications this has on the construct of your data set thus the outcomes.

Authors’ response: We have replied to the reviewer’s comment regarding the ICD-codes used for ascertainment of PND in the presented work and have added justification and details for the used ICD-10 codes to the manuscript.

We also cite the authors’ response to the reviewer’s comment below:

Authors’ response: The point is well taken. The aim is to capture common diagnoses indicating depressive disorders during defined perinatal period. The ICD codes used in the ascertainment of PND were chosen according to the classification of ICD-10 used in Swedish healthcare registers issued by the Swedish National Board of Health and Welfare [11]. Same codes were used in previous studies to identify perinatal depression among Swedish women using Swedish register data [12].

Specifically, F32 and F33 are ICD-10 codes used to identify depression in Swedish healthcare registers [11]; and have been reported to have high validity in the Swedish population (κ of 0·32; 88% full agreement with gold standard) [13]. F53.0 identifies depression occurred in perinatal period not captured elsewhere [11].

F34, F38 and F39 were not included for the following reasons:

- F34 in ICD-10 in Swedish healthcare registers captures mood disturbances that are not pronounced enough to be described as mild hypomanic or depressive episodes.
- F38 was defined as other affective disorders that do not justify classification to depression as they are neither sufficiently severe nor long-lasting.
- F39 captures affective psychosis.

Therefore, we used F32, F33 and F53.0 registered during the perinatal period, defined as from the date obtained by subtracting gestational age from the delivery date till one year postpartum, to identify women with PND. Rational for the choice of ICD-10 codes was revised as below:

Page 8, line 153-161: “In line with previous studies [12], we identified PND from the date obtained by subtracting gestational age from the delivery date till one year postpartum, using the Swedish version of International Statistical Classification of Diseases and Related Health Problems (ICD)-10 codes (F32, F33 and F53.0) recorded in the MBR and Patient Register. According to the Swedish National Board of Health and Welfare, F32 and F33 are ICD-10 codes used to identify depression in Swedish healthcare registers according to the Swedish National Board of Health and Welfare [11]; and have been reported to have high validity in the Swedish population (κ of 0·32; 88% full agreement with gold standard) [13]. F53.0 identifies perinatal depression not captured elsewhere [11].”

Nevertheless, we checked the open data from the National Board of Health and Welfare, in all women aged 20-39 years, 7,313 (3.6% of all depression spectrum diagnosis F32-F34 and F38-F39) women received a diagnosis of F34, 1,094 (0.5%) of F38, and 3,721 (1.8%) of F39 during 2001-2017[14]. Although these data are not necessarily about depression during perinatal period, it is likely in the present study we have only missed a small number of women with mild mood disturbances or affective disorders which is unlikely to bias our results. In fact, this misclassification is likely non-differential and would have attenuated the bidirectional association. Nevertheless, we have noted that we didn’t capture women with mood disturbances or affective disorders that are not sufficiently severe or long-lasting to be classified as depressive episodes in the “Discussion” as below:

“Discussion”, page 21, line 449-454: “Lastly, relying on the Patient Register, we would have missed cases with less severe symptomology and did not seek healthcare service. Moreover, with the ICD code we used to identify PND, we might have missed a small number of mood disturbances or affective disorders that are not sufficiently severe or long-lasting to be classified as depressive episodes. Our findings thus may not generalize well to women with mild PMDs or PND symptomology.”

5. Line 117 – what is ‘PDR’? Please define, apologies if I have missed it previously.

Authors’ response: PDR stands for Prescribed Drug Register. To avoid confusion, we have spelled it out when mentioned in the manuscript.

6. As for the abstract, please quantify the main results with 95% CIs and p values. When reporting p values please report as p<0.001 and where higher as p=0.002, for example. When a p value is given, please specify the statistical test used to determine it.

Authors’ response: We have added p values to the presented results, when applicable. The statistical test used was described in “Methods”, page 13, line 289-280: “The statistical significance was set at the nominal two-sided 5% level.”

7. Please separate upper and lower CI bounds with commas as opposed to hyphens to prevent confusion with reporting of negative values. Suggest reporting statistical information as follows, ‘(OR 4.58; 95% CI [4.28,4.90];p</=) to improve accessibility and clarity for the reader.

Authors’ response: We have revised the reported results according to the suggested format throughout.

8. Please define the length of follow up (eg, in mean, SD, and range).

Authors’ response: We have added description of the follow up including mean, standardized deviation and range in the “Results” section.

Page 15, line 311-313: “During a mean follow-up of 6.90 years (Standardized Deviation 4.31, range 0.003-17.50) from 6 months postpartum onwards, we identified 25,553 newly diagnosed cases of PMDs (4,227 among women with PND) in the matched cohort study.”

TABLES

1. Please provide a table showing the baseline characteristics of the study population in the main manuscript, this is currently placed in the supporting information.

Authors’ response: We have moved the characteristics table to the main manuscript.

2. Thank you for indicating that your analyses are adjusted and the factors which are adjusted for. To help facilitate transparent data reporting please also include unadjusted analyses for comparison.

Authors’ response: The point is well taken. We have added results before additional adjustment of covariates to all tables in the main manuscript. Additionally, we have added report of results before and after additional adjustment for the main findings in the “Results” section.

Page 14, Line 292-295: “PMDs were associated with a higher risk of subsequent PND (OR4.98, 95%CI [4.74,5.23]; p<0.001). Additional adjustment of demographic factors including country of birth, educational level, region of residence and cohabitation status slightly attenuated the observed association (OR 4.76, 95%CI [4.52,5.01]; p<0.001) (Table 2).

Page 15, line 313-317: “Compared to their matched controls, women with PND were at higher risk of PMDs (HR 2.00, 95%CI [1.93,2.07]; p<0.001). Adjustment for pregnancy characteristics, history of psychiatric disorders, together with demographic factors slightly attenuated the association (HR 1.81, 95%CI [1.74,1.88]; p<0.001) (Table 4).”

3. Throughout, as for the main manuscript. Where 95% CIs are reported please use commas as opposed to hyphens to separate upper and lower bounds.

Authors’ response: We have revised the reported 95% CIs accordingly throughout the manuscript.

4. Throughout, as for the main manuscript, where 95% CIs are reported please also report p values as <0.001 and where higher the exact p value as p= 0.002, for example. Where p values are reported in the footnote(s) please detail the statistical test used to determine them.

Authors’ response: We have added p values where 95% CIs are reported as suggested. The statistical test used to determine p values were described in the “Statistical analysis” section in the “Methods”.

Page 13, line 279-280: “The statistical significance was set at the nominal two-sided 5% level.”

DISCUSSION

1. Please temper the language in your opening paragraph, ‘…in this study we found…’ might be helpful.

Authors’ response: Thank you for the suggestion. We have revised the opening paragraph as suggested.

Page 16, line 338-340: “In the present study based on data from Swedish national registers, we found a bidirectional link between premenstrual disorders and perinatal depression, which was pronounced for prenatal and postnatal depression and slightly stronger for postnatal depression.”

2. Please present and organize the Discussion as follows: a short, clear summary of the article's findings; what the study adds to existing research and where and why the results may differ from previous research; strengths and limitations of the study; implications and next steps for research, clinical practice, and/or public policy; one-paragraph conclusion.

Authors’ response: After carefully reviewing the “Discussion”, we believe the content and structure fit well the required standards. Given that this is an observational study, and we try to refrain from over-interpretations of our findings, we keep the implications and next steps for research, clinical practice, and/or public policy brief and embedded in the conclusion. But we are open to accommodate if necessary.

3. Line 260 – ‘260 women without psychiatric history of .’ this sentence is incomplete, please revise

Authors’ response: Apologies for the incompleteness of the sentence. We have revised it as follows:

Page 16, line 340-341: “The bidirectional association was verified among women without a history of psychiatric disorders.”

4. Line 265 – ‘..the study is the first…’ claims of primacy can be risky, suggest, ‘to our knowledge’ or similar.

Authors’ response: Thanks for the suggestion. We have revised the sentence as below

Page 16, line 346-349: “Taking advantage of prospectively collected data in Swedish healthcare registers, the study is the first, to our knowledge, to demonstrate a positive association between PMDs and subsequent risk of PND.”

5. As above (for the introduction) please take care when describing/discussing/differentiating retrospective Vs prospective data collection and study design. Importantly, prospectively collected data often contributes to retrospective study designs.

Authors’ response: We have carefully reviewed the “Discussion” to ensure clarity and accuracy of prospective or retrospective data or design. Relevant contents in the “Discussion” are listed below:

Page 16, line 345-352: “Previous studies have used data on history of PMDs retrospectively collected during or after pregnancy, which might be vulnerable to recall bias and systematic error [2,3]. Taking advantage of prospectively collected data in Swedish healthcare registers, the study is the first, to our knowledge, to demonstrate a positive association between PMDs and subsequent risk of PND. Moreover, previous studies exclusively focused on postnatal depression, mostly ascertained within 8 weeks postpartum [4,5]. With retrospectively collected data, one systematic review and one meta-analysis reported that women with postnatal depression were more likely to endorse a history of PMDs [4,5], which is in line with our results.”

Page 19, line 415-417: “The strength of the study lies in the large sample size with long and complete follow-up, the prospectively and independently collected data on PMDs and PND, and covariates that could confound the studied association.”

6. Line 270 – please use a comma instead of a hyphen to separate upper and lower CI bounds. Please also indicate to the reader that the numerical values represent CIs. Please use formatting as detailed above under abstract and methods/results.

Authors’ response: We have revised the sentence accordingly.

7. Line 272 – perhaps ‘more than three times the risk’ instead, to improve accessibility for the reader.

Authors’ response: We have revised the sentence accordingly.

8. Your discussion of the study strengths could be more detailed. As above, in reference to the dual study design used to answer your questions what advantages does this offer? Please discuss.

Authors’ response: We appreciate your recognition of the study design adopted. The study design, a nested-case control study in combination with a cohort design, is certainly a strength of the study. This design allows us to study the association between PMDs and PND in a bidirectional fashion within the same population and with efficiency that we don’t need to do matching twice.

Page 19, line 415-420: “The strength of the study lies in the large sample size with long and complete follow-up, the prospectively and independently collected data on PMDs and PND, and covariates that could confound the studied association. The nested-case control study in combination with the transition into a matched cohort study with onward follow-up allowed us to study the association between PMDs and PND in a bidirectional fashion with efficiency, which is equivalent to two independent cohort studies within the same study population.”

9. Line 316 – please remove the subheading ‘Limitations’ such that the discussion reads as continuous prose.

Authors’ response: We have removed the subtitle as suggested.

10. Please remove the funding, disclosure and conflict statements form the main manuscript and include only in the manuscript submission form when you re-submit your manuscript. In the event of publication these will be compiled as metadata.

Authors’ response: We have removed the funding, disclosure and statement of conflict of interest from the manuscript and included them in the manuscript submission form instead.

REFERENCES

For in-text reference callouts please place citations in square brackets and preceding punctuation. For example [1,3]. Please note the absence of spaces between citations.

In the bibliography please list up to but no more than 6 author names followed by et al in that event that more than 6 contribute to the study.

Please ensure that web references include an ‘Accessed [date]’

Journal name abbreviations should be those found in the National Center for Biotechnology Information (NCBI) databases.

Please see our website for other reference guidelines <https://eur01.safelinks.protection.outlook.com/?url=https%3A%2F%2Fjournals.plos.org%2Fplosmedicine%2Fs%2Fsubmission-guidelines%23loc-references&data=05%7C01%7Cqian.yang.1%40ki.se%7C025150da3b334e88df5308dbd63565d2%7Cbff7eef1cf4b4f32be3da1dda043c05d%7C0%7C0%7C638339297198185909%7CUnknown%7CTWFpbGZsb3d8eyJWIjoiMC4wLjAwMDAiLCJQIjoiV2luMzIiLCJBTiI6Ik1haWwiLCJXVCI6Mn0%3D%7C3000%7C%7C%7C&sdata=7MWjok3QXrAB1TS8Lcum7sXoQUsepBaH763uTSV2O4s%3D&reserved=0>

Authors’ response: Thank you for the detailed instruction. We have updated the style of citations accordingly.

SUPPORTING INFORMATION

1. Please cite your Supporting Information as outlined here: <https://eur01.safelinks.protection.outlook.com/?url=https%3A%2F%2Fjournals.plos.org%2Fplosmedicine%2Fs%2Fsupporting-information&data=05%7C01%7Cqian.yang.1%40ki.se%7C025150da3b334e88df5308dbd63565d2%7Cbff7eef1cf4b4f32be3da1dda043c05d%7C0%7C0%7C638339297198185909%7CUnknown%7CTWFpbGZsb3d8eyJWIjoiMC4wLjAwMDAiLCJQIjoiV2luMzIiLCJBTiI6Ik1haWwiLCJXVCI6Mn0%3D%7C3000%7C%7C%7C&sdata=GhssDnV16%2BiNonhi8SkndPq1cGvOCKpBKpdydRXTz8w%3D&reserved=0>

Authors’ response: We have updated the citation of supporting information as instructed.

2. In the published article, supporting information files are accessed only through a hyperlink attached to the captions. For this reason, you must list captions at the end of your manuscript file. You may include a caption within the supporting information file itself, as long as that caption is also provided in the manuscript file. Do not submit a separate caption file.

Authors’ response: We have included captions of supporting information at the end of the manuscript.

Page 21-22, line 461-476:

“Supporting information

S Methods. Data origin and rational of the choice of covariates

S1 Fig. Flow chart

S1 Checklist

S1 Table. International Classification of Diseases codes used to define the studied medical conditions

S2 Table. Association of premenstrual disorders (PMDs) with subsequent risk of perinatal depression (PND) adjusted for mediators: a nested case-control study.

S3 Table. Stratified association of premenstrual disorders (PMDs) with subsequent perinatal depression (PND): a nested case control study.

S4 Table. Stratified association of perinatal depression (PND) with subsequent premenstrual disorders (PMDs): a matched cohort study.

S5 Table. Bidirectional link between perinatal depression (PND) with premenstrual disorders (PMDs): sensitivity analysis.

S6 Table. Association of perinatal depression (PND) with subsequent premenstrual disorders (PMDs): a matched cohort study with different start of follow-up.”

3. Please ensure that all abbreviations are defined for the reader at first use.

Authors’ response: We have defined all abbreviations at first use.

Please ensure that tables follow out guidance outlined above as for the main manuscript, including the provision of p values and unadjusted analyses, and use of commas to separate upper and lower CI bounds.

Authors’ response: We have updated all tables as requested.

As above, please include the completed STROBE checklist as Supporting Information. When completing the checklist, please use section and paragraph numbers, rather than page numbers as these often change in the event of publication. Please be reminded to add the following statement, or similar, to the Methods: "This study is reported as per the Strengthening the Reporting of Observational Studies in Epidemiology (STROBE) guideline (S1 Checklist)."

Authors’ response: We have completed the STROBE checklist and added the statement as required. Please refer to authors’ response to the editors’ general comment #2.

Please ensure that your supporting information follows the referencing guidance outlined above.

Authors’ response: Supporting information is referred as instructed. Please refer to authors’ response to the editors’ comment #1 and #2 in the “Supporting information” section.

Comments from the reviewers:

Reviewer #1: This is a well-conducted nationwide register-based study on the bidirectional association between premenstrual disorders and perinatal depression. The study design, datasets, statistical methods and analyses, and presentation (tables and figures) and interpretation of the results are mostly adequate and of a good standard. However, still a few issues needing attention.

Authors’ response: Thank you for your positive comments on our work.

1) There are 10 matched controls for each index case. Are these controls used for both the nested case-control study and the matched cohort study? or some used for each and at what split?

Authors’ response: After the matching, we first conducted a nested case-control study to examine the association between PMDs and subsequent risk of PND. All matched controls were used for the nested case-control study. We then transitioned the matched cases and controls into a matched cohort study with onward follow-up to examine the association between PND and subsequent risk of PMDs. We have clarified the study design in “Methods”:

Page 10, line 192-199: “To effectively examine the bidirectional association between PMDs and PND within the same study population, we conducted a nested case-control study, a design inherently equivalent to a cohort study [1], identifying all pregnancies, corresponding PNDs and previous history of any indications for PMDs to prospectively examine the risk of PND following PMDs. We then transitioned this design into a matched cohort with onward follow-up of index PNDs and control pregnancies, enabling us to efficiently assess the risk of PMDs after PND within the same study population.”

As we noted in the “Study design” in the “Methods”, page , line : In the matched cohort part, “Women who had PMDs before their index date were excluded (n=7,687) and, as they were not at risk for incident PMD diagnosis.”

2) For the Cox models in the matched cohort study, as the outcome is subsequent premenstrual disorders (PMDs) rather than all cause mortality, there is a potential competing risk issue, e.g., from death. Do we have mortality data for the study cohorts? Presumably low, but good to know.

Authors’ response: As we wrote in the “Study design” in the “Methods”, page 10, line 200-203: “All women were then followed from 6 months after delivery (by when over 90% of Swedish women had stopped complete breastfeeding) [15] or the index date, whichever came later, until age 52, emigration, death, PMD diagnosis, or end of follow-up, whichever came first.” Information on death was retrieved from the Cause of Death Register, as described in “Data sources” and women who died during the follow-up in the cohort study were right truncated.

We have also noted the number of death in our cohort in “Methods”, page 10, line 203-204: “During the follow-up, we observed 500 deaths in PND group and 1,559 deaths in matched controls.” As the reviewer has mentioned, the death number is small (0.2%) and the competing risk issue due to death, if any, in this population is unlikely to bias our results.

3) Missing data. There is no mention at all on missing data in the study. Are there any? How were the missing data dealt with?

Authors’ response: Like any other studies, there are observations with missing data. However, Swedish national healthcare registers are generally known for its extensive coverage and data of high quality. In particular, the Swedish Medical Birth Register and the Swedish Patient Register, which the submitted work primarily relied on, have been validated for its coverage and data quality [16,17].

Moreover, the missing rate for different variables may vary, naturally. The missing rate for each covariate included in the study was presented in Table 1, if any. As shown in Table 1, the missing rate for these covariates ranged from 0.0%-9.6%. Observations with missing values for the covariates included in the regression model were categorized as unknown. Given the low missing rate and big sample size in this nationwide register-based study, we consider the bias resulted from missing data minimal. Moreover, results before and after adjustment of the covariates were presented side by side in all tables, confirming consistently robust associations noted between PMDs and PND. Nevertheless, we have clarified how we handled missing data for covariates in “Methods”, page 11, line 219-220.

“ICD codes are summarized in S1 Table and categorization for covariates are presented in Table 1. Missing values in covariates were coded as unknown for adjustment.”

Reviewer #2: This paper is a significant contribution to knowledge of mood disorders affecting menstruating and pregnant people, and the authors should be congratulated for their careful 2-part design to characterize the bidirectional nature of the relationship between menstrually related mood disorders and perinatal depression.

Authors’ response: We thank the reviewer for the positive feedback on our study.

A minor comment for your consideration:

1. The paper in a couple of places conflates premenstrual disorders (PMD's, which is the basis for your analysis) and the DSM diagnosis of premenstrual dysphoric disorder (PMDD). The criteria you appear to be using for PMDs is more broad than that

Authors’ response: Indeed, DSM has listed diagnostic criteria for PMDD while the mild type, premenstrual syndrome (PMS) was defined by the American College of Obstetricians and Gynecologists (ACOG). We have revised the “Ascertainment of PMDs” accordingly.

Page 9, line 175-181: “PMDs include premenstrual syndrome (PMS) and premenstrual dysphoric disorder (PMDD). In practice, PMS is often diagnosed based on criteria similar to the American College of Obstetricians and Gynecologists (ACOG) criteria [18], and PMDD are diagnosed in accordance with diagnostic criteria described in DSM-5 [19]. According to the Swedish guidelines, prospective daily symptom ratings for at least two consecutive menstrual cycles are required [20].

the Lancet article you reference (3) is about premenstrual syndrome and refers to findings of prospective and retrospective studies suggest that 5-8% of women with hormonal cycles have moderate to severe symptoms, not the 20-30% you state in this article.

Authors’ response: Thank you for pointing out the error. We have carefully cross-checked with the cited paper and revised as below.

Page 6, line 109-110: “PMDs affect 20-30% of women of reproductive age [21], and about 5–8% of women suffer from severe symptomology [22].”

In addition, you refer to the Swedish guidelines using DSM-5 criteria requiring prospective rating. However, DSM-5 was first published in 2013, and your study starts in 2001. In your previous paper on PMDs and injury (ref 4) you more accurately characterize that your PMD case ascertainment is not clearly based on prospective rating. You should clarify this distinction in this paper similarly - as written it is misleading to the reader.

Authors’ response: Thank you for noting this. We have enriched the writing on this point and discussed the validity of PMD diagnosis in our data thoroughly and how the changes of labeling and diagnostic criteria for PMDs could impact our results in the “Discussion”.

Page 20, line 422-435: “First, the clinical diagnosis of PMDs in the Swedish Patient Register has not been validated. Although prospective daily symptom ratings for at least two consecutive menstrual cycles are required for diagnosing PMDs in Sweden according to the guidelines [20], we cannot confirm that the clinical decision to assign a diagnosis or medical treatment to every single ascertained PMD in the registers is based on prospective daily ratings. However, clinical guidelines are often well followed owing to the state-funded nature of the public healthcare system in Sweden. Moreover, the Swedish Patient Register has fairly high validity in general [23], with the overall positive predicted value for most diagnoses ranging from 85%- 95% [24]. For a range of psychiatric disorders [25-28] and gynecologic diseases [17,29], the diagnosis has been reported to have high validity. Lastly, sensitivity analysis restricting to PMDs with at least two specialist-made diagnoses at least 28 days apart yielded similar results. In addition, the diagnostic criteria for PMDs may have changed over time. However, stratification analysis by calendar year showed similar results, suggesting that our results are robust given the changes of labeling and diagnostic criteria for PMDs over time.”

Reviewer #3

Thank you for the opportunity to review this important piece of work on the bidirectional relationship between premenstrual disorders (PMDs) and perinatal depression (PND). I have provided detailed comments in the order of the main text sections. Analyses performed in this study is well presented however, the writing and logic of this paper warrants improvement. A major revision is my recommendation for the decision of publication of this paper in PLoS Medicine.

Authors’ response: Thank you for your recognition of the analyses conducted in this study and for your detailed comments.

Introduction

1. Line 61, ‘Similarly, premenstrual disorders (PMDs) are characterized by … ’: please provide basic background knowledge about PMDs as ‘core PMDs’ is the term recently defined in the RCOG premenstrual disorder guideline.

Authors’ response: Thanks for the suggestion. We have revised the introduction of PMDs in the “Background” based on characteristics of “core premenstrual disorder” defined by ISPMD consensus and included the citation in the manuscript.

Page 6, line 107-109: “Similarly, premenstrual disorders (PMDs) are characterized by somatic and/or psychological symptoms that recur in luteal phase. PMDs cause significant functional impairment [21,22,30].”

2. Please add in a paragraph detailing the importance of researching the bidirectional relationship between PMD and PND. This echoes to what was illustrated in the Discussion section.

Authors’ response: Thank you for the suggestion. We have enriched the introduction of the study design in the “Introduction”.

Page 7, line 127-135: “Taken together, without prospective evidence, it remains unclear whether women with PMDs have an increased risk of developing PND when becoming pregnant or after giving birth, and vice versa. Using the Swedish nationwide registers with prospectively collected data, we aimed to investigate the bidirectional association between PMDs and PND. To study the bidirectional association in the same population effectively, we conducted a nested case-control study, a design inherently equivalent to a cohort study [1], to prospectively examine the risk of PND following PMDs. We then transitioned this design into a matched cohort study to assess the risk of PMDs after PND. We further employed sibling comparisons to account for shared genetic and familial environmental risk factors for both disorders.”

3. Line 69, ‘However, existing studies had retrospective design, …’: This is not for all previous studies – many of them adopted a prospective study design. The biggest advantage of this present study is that it uses the Swedish data registry which is a population-based sampling that to a large extend reduced the selection bias compared to any other previous studies.

Authors’ response: Indeed, some studies included in the systematic reviews claimed prospective design in the report. Data on premenstrual symptoms were, however, retrospectively collected during and shortly after pregnancy, of which the assessment of premenstrual symptoms (absent during perinatal period) might be prone to recall bias. We have revised the sentence to be more accurate.

We agree with the reviewer that community- or clinic-based sampling in previous studies may have introduced selection bias while the nationwide population-based data used in the presented work has to a large extent reduced the selection bias. We have noted this point in the “Introduction”.

Page 7, line 117-120: “However, existing studies relied on retrospectively collected on data premenstrual symptoms during or after pregnancy, which might be prone to recall bias and thereby biased results [2,3]. Moreover, the community- or clinic-based sampling in previous studies may have introduced significant selection bias [6].”

4. Line 73, ‘no study has examined this hypothesis’: this statement is simply not true. Studies on the same topic have already been carried out in the 80s. See refs:

- Dennerstein L, Morse CA, Varnavides K. Premenstrual tension and depression — is there a relationship? Journal of Psychosomatic Obstetrics & Gynecology. 1988;8(1):45-52.
- Warner P, Bancroft J, Dixson A, Hampson M. The relationship between perimenstrual depressive mood and depressive illness. Journal of Affective Disorders. 1991;23(1):9-23.
- Haywood A, Slade P, King H. Is there evidence of an association between postnatal distress and premenstrual symptoms? Journal of Affective Disorders. 2007;99(1-3):241-245.

Authors’ response: Thank you for noting this. We have acknowledged the shared publications in the manuscript as below:

Page 6-7, line 122-125: “It is thus plausible that women with PND are at risk for subsequent PMDs. However, few studies with a relatively small sample size have examined this hypothesis by comparing proportions without adjustment for confounders and generated inconsistent results [8-10].”

Methods

1. Ascertainment of PND

- Line 95, ‘in line with previous studies [ref 18], we identified PND from … using ICD-10 codes (F32, F33 and F59.0)’. This sentence is misleading as the paper being cited used a different set of ICD-10 codes to infer PND. Also, another paper the authors cited below ‘the diagnosis of major depression with used ICD codes has been validated in the Patient Register’ seems to be the right reference for this argument. After checking, their study only used ICD-10 codes F32-F33.9, which is also different the present study. Please correct. In addition, please justify why ICD-10 codes like F53, F34, F38 and F39 were not used to infer PND diagnoses. They are all frequently use for the ascertainment of PNDs [ref: Xu F, Austin M-P, Reilly N, et al. Archives of Women’s Mental Health. 2012;15(5):333.]

Authors’ response: The point is well taken. The aim is to capture common diagnoses indicating depressive disorders during defined perinatal period. The ICD codes used in the ascertainment of PND were chosen according to the classification of ICD-10 used in Swedish healthcare registers issued by the Swedish National Board of Health and Welfare [11]. Same codes were used in previous studies to identify perinatal depression among Swedish women using Swedish register data [12].

Specifically, F32 and F33 are ICD-10 codes used to identify depression in Swedish healthcare registers [11]; and have been reported to have high validity in the Swedish population (κ of 0·32; 88% full agreement with gold standard) [13]. F53.0 identifies depression occurred in perinatal period not captured elsewhere [11].

F34, F38 and F39 were not included for the following reasons:

- F34 in ICD-10 in Swedish healthcare registers captures mood disturbances that are not pronounced enough to be described as mild hypomanic or depressive episodes.
- F38 was defined as other affective disorders that do not justify classification to depression as they are neither sufficiently severe nor long-lasting.
- F39 captures affective psychosis.

Therefore, we used F32, F33 and F53.0 registered during the perinatal period, defined as from the date obtained by subtracting gestational age from the delivery date till one year postpartum, to identify women with PND. Rational for the choice of ICD-10 codes was revised as below:

Page 8, line 153-161: “In line with previous studies [12], we identified PND from the date obtained by subtracting gestational age from the delivery date till one year postpartum, using the Swedish version of International Statistical Classification of Diseases and Related Health Problems (ICD)-10 codes (F32, F33 and F53.0) recorded in the MBR and Patient Register. According to the Swedish National Board of Health and Welfare, F32 and F33 are ICD-10 codes used to identify depression in Swedish healthcare registers according to the Swedish National Board of Health and Welfare [11]; and have been reported to have high validity in the Swedish population (κ of 0·32; 88% full agreement with gold standard) [13]. F53.0 identifies perinatal depression not captured elsewhere [11].”

Nevertheless, we checked the open data from the National Board of Health and Welfare, in all women aged 20-39 years, 7,313 (3.6% of all depression spectrum diagnosis F32-F34 and F38-F39) women received a diagnosis of F34, 1,094 (0.5%) of F38, and 3,721 (1.8%) of F39 during 2001-2017[14]. Although these data are not necessarily about depression during perinatal period, it is likely in the present study we have only missed a small number of women with mild mood disturbances or affective disorders which is unlikely to bias our results. In fact, this misclassification is likely non-differential and would have attenuated the bidirectional association. Nevertheless, we have noted that we didn’t capture women with mood disturbances or affective disorders that are not sufficiently severe or long-lasting to be classified as depressive episodes in the “Discussion” as below:

“Discussion”, page 21, line 449-454: “Lastly, relying on the Patient Register, we would have missed cases with less severe symptomology and did not seek healthcare service. Moreover, with the ICD code we used to identify PND, we might have missed a small number of mood disturbances or affective disorders that are not sufficiently severe or long-lasting to be classified as depressive episodes. Our findings thus may not generalize well to women with mild PMDs or PND symptomology.”

- Line 99, ‘although such definition of PND was not specifically validated, the diagnosis of major depression with used ICD codes has been validated in the Patient Register’: What does the result say?

Authors’ response: Please refer to the authors’ response to the comment above.

2. Study design

- Line 130: Please explain what ‘the diagnosis/matching date’ means in detail. Why not simply state it as ‘matching date’? I assume no matter prenatal or postnatal depression, cases were matched on the diagnosis date by the gestational age for prenatal and postnatal day, respectively.

Authors’ response: Thank you for the suggestion. We have revised as suggested.

Page 10, line 199: “The matching date was used as the index date.”

3. Statistical analysis

- Line 158, ‘Due to the design, the OR can be interpreted as HR…’: please elaborate on this using one or two sentences for the wider audience who is unfamiliar with epidemiology.

Authors’ response: The point is well taken.

Page 11, line 229-233: “The nested case–control analysis, by design, is equivalent to the analysis using full cohort (i.e., assessing the incidence of PND subsequent to PMDs). Using incidence density sampling for individual matching, the estimate OR can be interpreted as hazard ratio (HR) of PND, comparing women with and without PMDs before pregnancy [1].”

- Line 168, ‘stratified analysis by maternal age … ’: What was the relationship between maternal age (continuous variable) and PND? Which table shows this result?

Authors’ response: “To explore effect modification by maternal age and calendar year, we also conducted stratified analysis by maternal age (categorized into 15-30 and 31-52) and calendar year at pregnancy.” (page 12, line 241-243). Results from the stratification analysis by maternal age are referred to in the “Results”.

“PMDs and subsequent risk of PND”

Page 14-15, line 306-308: “The association was stronger among multiparous than primiparous women and was comparable across maternal age and calendar year groups (S3 Table).”

“PND and subsequent risk of PMDs”

Page 15, line 322-324: “The association did not differ by parity and calendar year and was somewhat greater among women aged 31-52 years at pregnancy (S4 Table).”

- Line 170, ‘PND and subsequent risk of PMDs’: please explain why different study design were sued for the two research questions. Why not use matched cohort to explore PMDs🡪PND as well or use nested case-control for PND🡪PMDs?

Authors’ response: Thank you for the suggestion. Although we used different study designs for two research questions, the advantage is that we only need to do matching once.

We have clarified the study design in the “Methods”, page 10, line 192-199: “To effectively examine the bidirectional association between PMDs and PND within the same study population, we conducted a nested case-control study, a design inherently equivalent to a cohort study [1], identifying all pregnancies, corresponding PNDs and previous history of any indications for PMDs to prospectively examine the risk of PND following PMDs. We then transitioned this design into a matched cohort with onward follow-up of index PNDs and control pregnancies, enabling us to efficiently assess the risk of PMDs after PND within the same study population.”

- Line 175, ‘PND subtype’: add in brackets (i.e., those with and without a history of psychiatric disorders) if that is what the authors meant.

Authors’ response: We have clarified PND subtypes as below:

Page 12, line 249-251: “Consistent with the nested case-control analyses, we performed analyses by PND subtype (pre- and postnatal depression; and PND diagnosed in different time windows) and other stratification analyses.”

Results

1. PMDs and subsequent risk of PND

- Line 226, ‘was stronger among women without such history’: this result is very counter-intuitive and new findings as such (new finding no. 1) warrants a thorough discussion.

Authors’ response: Thank you for the suggestion. The reviewer is right that the absolute risk of PND is higher among women with a history of psychiatric disorder. However, the association (relative risk, i.e., OR and HR) was stronger among women without a history of psychiatric disorders. A plausible reason to the greater relative risk observed among women without a history of psychiatric disorders is that the absolute risk is lower in the reference group without a history of psychiatric disorders as the denominator, which results in a higher relative risk. It is easier to illustrate this in the matched cohort study. For instance, as the denominator, the incidence rate of PMDs in the reference group without a history of psychiatric disorders is 3.7 per 1000 person-years, as opposed to 8.4 per 1000 person-years in the reference group with a history of depression (currently Table 5). That said, in women without psychiatric history, PND confers a 2-fold higher risk of subsequent PMDs (HR=2.08). However, among those with a history of depression which is also associated with a higher risk of PMDs, the PND does not confer any additionally increased risk of subsequent PMDs (HR=0.94).

To avoid confusion, we have clarified this in the discussion as shown below:

“Discussion” (page 18, line 386-397), “First, both PMDs and PND have shared liability with psychiatric disorders [31,32]. which may explain the findings. PMDs could also lead to the development of psychiatric comorbidities, which increase the risk of PND and *vice versa* [31,32]. In this scenario, psychiatric disorders are mediators of the studied associations. Indeed, additional adjustment of psychiatric disorders attenuated the associations. However, the associations (relative risks) remained robust and even stronger among women without a history of psychiatric disorders, suggesting our findings cannot be entirely explained by psychiatric disorders. On the other hand, although the absolute risk (e.g., probability or incidence rate) of PMDs is higher among women with a psychiatric history, a diagnosis of PND does not translate into an increased risk of PMDs to the same extent as it does for those without a psychiatric history. This is likely because the already heightened risk of PMDs among those with a psychiatric history.”

- Line 227, the association was also stronger among multiparous than primiparous women: same for this new finding (new finding no. 2) too.

Authors’ response: Great point. We have added discussion on this finding.

“Discussion”, page 17, line 363-369: “Interestingly, we found a stronger association between PMDs and PND among multiparous than primiparous women. It is known that primiparous women have a higher risk of PND [33] while parity is positively associated with PMDs [34]. It is plausible that premenstrual symptoms can worsen after pregnancy likely due to an escalated abnormal response to hormonal changes in relation to pregnancy [7]. However, it is unclear whether multiple pregnancies would further deteriorate the pathological response to hormone fluctuations. Future studies are needed to better understand the potential mechanisms.”

2. PND and subsequent risk of PMDs

- PND🡪PMDs this association stronger in those without history of psychiatric disorders too: new finding worth discussing (new finding no. 3)

Authors’ response: Please refer to the authors’ response to the comment above.

- Line 241, Table 4: how to explain also positive results among those without history of other psychiatric disorders (new finding no. 4)?

Authors’ response: “the associations remained robust among women without psychiatric disorders, suggesting our findings cannot be entirely explained by psychiatric disorders.” (“Discussion”, page 18, line 391-393)

For more details, please refer to the authors’ response to the comment above.

Discussion

1. Line 273, ‘to our knowledge, no study has … the association between prenatal depression and PMDs’: yes, but please expand a bit on why this is important/relevant.

Authors’ response: Previous studies have primarily focused on postpartum depression, the observed bidirectional association between prenatal depression and PMDs therefore extends the knowledge to prenatal depression, and may highlight a subgroup of prenatal depression that is associated with hormone fluctuations.

“Discussion”, page 17, line 358-363: “To our knowledge, no study has examined the association between prenatal depression and PMDs. Our findings on prenatal depression therefore extend knowledge to another subtype of PND that occurs during pregnancy. Although the etiology for prenatal depression is complex [35], our finding may suggest a subgroup of prenatal depression may be related to hormone changes as well. However, future studies are needed to confirm our results and to understand the biologic link between PMDs and prenatal depression.”

2. Lines 281-286, ‘likely to take antidepressants, which may mitigate premenstrual symptoms’: I assume there are analyses of this paper that have accounted for this. E.g., to adjust for antidepressants use prior to the outcome.

Authors’ response: We agree with the reviewer that it’s of research interest to investigate how the use of antidepressants for PMDs or PND would modify the association between PMDs and PND. However, use of antidepressants is a time-varying exposure and the effect of usage of antidepressants can be confounded by indication bias. Therefore, a separate study is needed to carry out a full investigation on this research question.

Moreover, the section was to discuss the stronger association between PMDs and subsequent PND then the association in the other direction. We have revised the section as below to avoid misunderstanding.

“Discussion”, page 18, line 376-384: “Interestingly, we noted a stronger association between PMDs and subsequent PND than the association in the other direction. This might be because many PMDs have an early onset [36], likely before the average age at first childbirth, whereas we targeted the late-onset PMDs in the cohort study. It is also plausible that women with PND are more likely to take antidepressants, which may mitigate premenstrual symptoms. On the other direction, although women with PMDs may use antidepressants as well, women are more likely to discontinue psychotic medications during pregnancy and even after due to breastfeeding [37,38]. However, future studies are warranted to disentangle the role of treatment in the differential associations observed for both directions.”

Reference

1.	Ernster, V.L., *Nested case-control studies.* Prev Med, 1994. **23**(5): p. 587-90.

2.	Battle, D.E., *Diagnostic and Statistical Manual of Mental Disorders (DSM).* Codas, 2013. **25**(2): p. 191-2.

3.	ACOG. *Premenstual Syndrome (PMS)*. May, 2015; Available from: <https://www.acog.org/womens-health/faqs/premenstrual-syndrome>.

4.	Cao, S., et al., *History of premenstrual syndrome and development of postpartum depression: A systematic review and meta-analysis.* J Psychiatr Res, 2020. **121**: p. 82-90.

5.	Amiel Castro, R.T., E.A. Pataky, and U. Ehlert, *Associations between premenstrual syndrome and postpartum depression: A systematic literature review.* Biol Psychol, 2019. **147**: p. 107612.

6.	Kleinbaum, D.G., H. Morgenstern, and L.L. Kupper, *Selection bias in epidemiologic studies.* Am J Epidemiol, 1981. **113**(4): p. 452-63.

7.	Chuong, C.J. and D.M. Burgos, *Medical history in women with premenstrual syndrome.* J Psychosom Obstet Gynaecol, 1995. **16**(1): p. 21-7.

8.	Warner, P., et al., *The relationship between perimenstrual depressive mood and depressive illness.* J Affect Disord, 1991. **23**(1): p. 9-23.

9.	Haywood, A., P. Slade, and H. King, *Is there evidence of an association between postnatal distress and premenstrual symptoms?* J Affect Disord, 2007. **99**(1-3): p. 241-5.

10.	Dennerstein, L., C.A. Morse, and K. Varnavides, *Premenstrual tension and depression - is there a relationship?* Journal of psychosomatic obstetrics and gynaecology, 1988. **8**(1): p. 45-52.

11.	Welfare, t.S.N.B.o.H.a., *Klassifikationer.* updated 2023-01-01.

12.	Naela Hagatulah, E.B., Anna Sara Oberg, Unnur A. Valdimarsdóttir, Qing Shen, Donghao Lu, *Perinatal depression and risk of mortality: a nationwide register-based study in Sweden.* BMJ in press, 2023.

13.	Fazel, S., et al., *Depression and violence: a Swedish population study.* Lancet Psychiatry, 2015. **2**(3): p. 224-32.

14.	Sweden, S. *Publiceringskalender för statistik*. 2023 2023-10-18 [cited 2023 2023-11-15]; Available from: <https://www.socialstyrelsen.se/statistik-och-data/statistik/publiceringskalender-for-statistik/>.

15.	Socialstyrelsen. *Statistics on breastfeeding* 2021-05-25 [cited 2022 2022-03-28]; Available from: <https://www.socialstyrelsen.se/statistik-och-data/statistik/alla-statistikamnen/amning/>.

16.	Cnattingius, S., et al., *The Swedish medical birth register during five decades: documentation of the content and quality of the register.* Eur J Epidemiol, 2023. **38**(1): p. 109-120.

17.	Ludvigsson, J.F., et al., *External review and validation of the Swedish national inpatient register.* BMC Public Health, 2011. **11**: p. 450.

18.	(ACOG), t.A.C.o.O.a.G. *Premenstual Syndrome (PMS)*. 2015 2023-10-31]; Available from: <https://www.acog.org/womens-health/faqs/premenstrual-syndrome>.

19.	Feingold KR, A.B., Blackman MR, et al., *Table 1Diagnostic Criteria for Premenstrual Dysphoric Disorder (PMDD).*

20.	förlossning, R.S.l.e.f.k.o., *Riktlinjer vid premenstruell dysforisk störning, PMDS.* 2023-03-13.

21.	Yonkers, K.A. and M.K. Simoni, *Premenstrual disorders.* Am J Obstet Gynecol, 2018. **218**(1): p. 68-74.

22.	Yonkers, K.A., P.M. O'Brien, and E. Eriksson, *Premenstrual syndrome.* Lancet, 2008. **371**(9619): p. 1200-10.

23.	Forsberg L, R.H., Jacobsson A, Nyqvist K, Heurgren M, *Kvalitet och innehåll i patientregistret. Utskrivningar från slutenvården 1964-2007 och besök i specialiserad öppenvård (exklusive primärvårdsbesök) 1997-2007. (Quality and content of the Patient Register)(2009-125-15)*, in *Kvalitet och innehåll i patientregistret. Utskrivningar från slutenvården 1964-2007 och besök i specialiserad öppenvård (exklusive primärvårdsbesök) 1997-2007. (Quality and content of the Patient Register)(2009-125-15)*. 2009: Stockholm.

24.	Chang, Z., et al., *Psychiatric disorders and violent reoffending: a national cohort study of convicted prisoners in Sweden.* Lancet Psychiatry, 2015. **2**(10): p. 891-900.

25.	Kendler, K.S., et al., *Genetic and family and community environmental effects on drug abuse in adolescence: a Swedish national twin and sibling study.* Am J Psychiatry, 2014. **171**(2): p. 209-17.

26.	Fazel, S., et al., *Schizophrenia, substance abuse, and violent crime.* JAMA, 2009. **301**(19): p. 2016-23.

27.	Sellgren, C., et al., *Validity of bipolar disorder hospital discharge diagnoses: file review and multiple register linkage in Sweden.* Acta Psychiatr Scand, 2011. **124**(6): p. 447-53.

28.	Ekholm, B., et al., *Evaluation of diagnostic procedures in Swedish patients with schizophrenia and related psychoses.* Nord J Psychiatry, 2005. **59**(6): p. 457-64.

29.	Ros, H.S., S. Cnattingius, and L. Lipworth, *Comparison of risk factors for preeclampsia and gestational hypertension in a population-based cohort study.* Am J Epidemiol, 1998. **147**(11): p. 1062-70.

30.	Nevatte, T., et al., *ISPMD consensus on the management of premenstrual disorders.* Arch Womens Ment Health, 2013. **16**(4): p. 279-91.

31.	Pilver, C.E., D.J. Libby, and R.A. Hoff, *Premenstrual dysphoric disorder as a correlate of suicidal ideation, plans, and attempts among a nationally representative sample.* Soc Psychiatry Psychiatr Epidemiol, 2013. **48**(3): p. 437-46.

32.	Beck, C.T., *Predictors of postpartum depression: an update.* Nurs Res, 2001. **50**(5): p. 275-85.

33.	Iwata, H., et al., *Prevalence of postpartum depressive symptoms during the first 6 months postpartum: Association with maternal age and parity.* J Affect Disord, 2016. **203**: p. 227-232.

34.	Sylven, S.M., et al., *Premenstrual syndrome and dysphoric disorder as risk factors for postpartum depression.* Acta Obstet Gynecol Scand, 2013. **92**(2): p. 178-84.

35.	Samuelsen, K., et al., *Kind of blue - An evaluation of etiologies for prenatal versus postnatal depression symptoms.* J Affect Disord, 2023. **335**: p. 305-312.

36.	Lu, D., et al., *Pubertal development and risk of premenstrual disorders in young adulthood.* Hum Reprod, 2021. **36**(2): p. 455-464.

37.	Einarson, A., P. Selby, and G. Koren, *Abrupt discontinuation of psychotropic drugs during pregnancy: fear of teratogenic risk and impact of counselling.* J Psychiatry Neurosci, 2001. **26**(1): p. 44-8.

38.	Molenaar, N.M., M.P. Lambregtse-van den Berg, and G.J. Bonsel, *Dispensing patterns of selective serotonin reuptake inhibitors before, during and after pregnancy: a 16-year population-based cohort study from the Netherlands.* Arch Womens Ment Health, 2020. **23**(1): p. 71-79.
