# Supplementary material for: The bidirectional association between premenstrual disorders and perinatal depression: A nationwide register-based study from Sweden
Source: PLoS Med. 2024 Mar 28;21(3):e1004363. doi: 10.1371/journal.pmed.1004363 (PMC10978009; doi:10.1371/journal.pmed.1004363)
Supplement: Attachment — Submitted filename: Response letter.docx [file pmed.1004363.s012.docx]

Manuscript ID: PMEDICINE-D-23-01426R3

Dear Dr. Dodd,

Thank you for the thorough review of our paper entitled “**The bidirectional association between premenstrual disorders and perinatal depression: a nationwide register-based study from Sweden**” and for the opportunity to publish our paper in *PLOS Medicine* provided that editorial requirements and comments are addressed*.*

We appreciate the detailed instructions on required revisions from the editor and the positive remarks from the reviewers and their acknowledgement of our work to address their comments. Please find our point-by-point responses to those comments below. We have revised the manuscript in accordance with the comments from the editor and the reviewers, including clarifying study design, reformatting references, and enriching discussion on important aspects.

We believe that the quality of the manuscript has, as a result, been significantly improved. While we are open to make further revisions, we hope that you will find the present version of our manuscript satisfactory and suitable for publication in *PLOS Medicine*.

All authors have contributed to this revision and approved the final version of the manuscript.

Please do not hesitate to contact us if further modifications are needed.

Yours sincerely,

Qian Yang and Donghao Lu

On behalf of all co-authors

Institute of Environmental Medicine, Karolinska Institutet

Nobels väg 13, S-17 177 Stockholm, Sweden

*All page and line marks correspond to the line numbers of the ‘Revised manuscript_clean version’.

COMMENTS FROM THE EDITORS:
GENERAL
Thank you for your detailed and considered responses to previous editor and reviewer comments. Please see below for further comments that we require you address prior to publication.

Authors’ response: Thank you for your acknowledgement of our work.

AUTHOR SUMMARY
Thank you for including an author summary which reads very nicely but is too long. Each sub-heading should precede 2-3 concise (1-2 sentence) bullet point statements (much of the statistical information could be removed to conserve space, for example). Please keep in mind that the summary should be accessible to a wide audience including the lay person. Please revise for brevity, in mind of the below guidance:

The author summary should consist of 2-3 succinct bullet points under each of the following headings:
• Why Was This Study Done? Authors should reflect on what was known about the topic before the research was published and why the research was needed.
• What Did the Researchers Do and Find? Authors should briefly describe the study design that was used and the study’s major findings. Do include the headline numbers from the study, such as the sample size and key findings.   
• What Do These Findings Mean? Authors should reflect on the new knowledge generated by the research and the implications for practice, research, policy, or public health. Authors should also consider how the interpretation of the study’s findings may be affected by the study limitations. In the final bullet point of ‘What Do These Findings Mean?’, please describe the main limitations of the study in non-technical language.

This text should be distinct from the scientific abstract. Please see our author guidelines for more information: <https://eur01.safelinks.protection.outlook.com/?url=https%3A%2F%2Fjournals.plos.org%2Fplosmedicine%2Fs%2Frevising-your-manuscript%23loc-author-summary&data=05%7C02%7Cqian.yang.1%40ki.se%7C1a5e0612cc4d47843aa908dbffe97dc7%7Cbff7eef1cf4b4f32be3da1dda043c05d%7C0%7C0%7C638385150525725491%7CUnknown%7CTWFpbGZsb3d8eyJWIjoiMC4wLjAwMDAiLCJQIjoiV2luMzIiLCJBTiI6Ik1haWwiLCJXVCI6Mn0%3D%7C3000%7C%7C%7C&sdata=3fpjd95PVm5M%2BMRwfP8bZoLiPyz9GJyto1TukWk28QM%3D&reserved=0> and please see our website for published examples <https://eur01.safelinks.protection.outlook.com/?url=https%3A%2F%2Fjournals.plos.org%2Fplosmedicine%2F&data=05%7C02%7Cqian.yang.1%40ki.se%7C1a5e0612cc4d47843aa908dbffe97dc7%7Cbff7eef1cf4b4f32be3da1dda043c05d%7C0%7C0%7C638385150525725491%7CUnknown%7CTWFpbGZsb3d8eyJWIjoiMC4wLjAwMDAiLCJQIjoiV2luMzIiLCJBTiI6Ik1haWwiLCJXVCI6Mn0%3D%7C3000%7C%7C%7C&sdata=3ddr%2B8jD0h%2BE4MtKJe2X5zuSRijwjiqrVQ0gJJfBCy4%3D&reserved=0>

Authors’ response: Thank you for the instruction. We have now revised the “Author summary” as below so it’s more concise and intuitively clear.

**Author summary**

**Why was this study done?**

- Perinatal depression (PND) and premenstrual disorders (PMDs) share symptomology (e.g., feeling depressed) and the timing of symptom onset of both conditions coincides with natural hormonal fluctuations.
- Prospective data are lacking to study the potential bidirectional association between these conditions, which can guide clinical management.

**What did the researchers do and find?**

- Among around 1.8 million singleton pregnancies in Sweden during 2001-2018, we identified 84,949 women of PND and 849,482 unaffected women, individually matched on age and calendar year. A nested case-control study and a matched cohort study were conducted to examine the bi-directional association between PMDs and PND. Pregnancies from full sisters of PND cases were also identified for sibling comparison.
- Among women with PND, 2.9% had PMDs before pregnancy, in contrast to 0.6% among matched unaffected women. PMDs were associated with a nearly five times higher risk of subsequent PND. In the matched cohort with a mean follow-up of 6.90 years, women with PND had almost two times higher risk of subsequent PMDs, compared to their matched unaffected women.
- The bidirectional association between PMDs and PND was noted for both prenatal and postnatal depression, regardless of history of psychiatric disorders, and also in sibling comparison.

**What do these findings mean?**

- These findings suggest that a history of PMDs can inform PND susceptibility and *vice versa*.

ABSTRACT
Line 32 – We agreed that case-control design being “equivalent to a cohort study but with improved efficiency” is not an accurate description. Please remove this statement.
We suggest, ‘…transitioned that design into a matched cohort study with onward follow-up to simulate a prospective study design and examine the risk of PMDs after PND…’. Please also see below for the same/similar elsewhere and amend accordingly and consistently throughout as necessary.

Authors’ response: Thank you for your suggestion. We have revised accordingly.

“Methods and findings” in the “Abstract”: “With 1,803,309 singleton pregnancies of 1,041,419 women recorded in the Swedish Medical Birth Register during 2001-2018, we conducted a nested case-control study to examine the risk of PND following PMDs, which is equivalent to a cohort study; and transitioned that design into a matched cohort study with onward follow-up to simulate a prospective study design and examine the risk of PMDs after PND (within the same study population).”

METHODS and RESULTS
Line 196 – ‘…to prospectively examine the risk…’ we understand what you mean but it is technically incorrect as all data is examined (in your study) in retrospect. Please revise suggest (as above), ‘to examine the risk…in a manner which simulates a prospective approach’

Authors’ response: We have revised accordingly as below.

Page 7, line 114-117: “To study the bidirectional association in the same population effectively, we conducted a nested case-control study, a design inherently equivalent to a cohort study [1], to examine the risk of PND following PMDs in a manner which simulates a prospective approach.”

TABLES
Table 1 – it would be worth indicating whether there are significant differences between the PND and control groups? Please include p values reporting a p<0.001 and where higher the exact p value as 0.002, for example.

Authors’ response: We have added p value in Table 1 as suggested. Because of the large sample size, all P values, except for the matching factors, are <0.001 even for small differences. We have made corresponding changes in the Results.

“Characteristics” in “Results”, page 14, line 270-275: Compared to the controls, women with PND were less educated, were more likely to be born in Sweden, live alone and in South Sweden, had a higher BMI and were more likely to smoke and have been diagnosed with psychiatric disorder before the pregnancy (all *P* values<0.001; Table 1). They were also more likely to be primiparous, deliver through cesarean section, and experience pregnancy complications and adverse pregnancy and birth outcomes (all *P* values<0.001; Table 1).

Tables 2-6 – please separate upper and lower CI bounds with commas as opposed to hyphens as the latter can be confused with reporting of negative values. Please include the statistical test used to determine p values in the footnotes.

Authors’ response: We have revised the format and added the footnote as required.

DISCUSSION
Thank you for your consideration of not overstating your findings. We do think that wider discussion of the implications of your study (in context of the limitations you highlight) and next steps for research would be helpful. How does this study specifically inform subsequent?

Authors’ response: Thank you for the suggestion. We have added a line for future studies in context of the limitations of the current study as below:

Page 21, line 446-449: “Preconception and maternity care providers should be aware of the risk of developing PND among women with a history of PMDs. Moreover, healthcare providers may inform PND women about the potential risk of PMDs when menstruation returns after childbirth.”

Lines 428 onwards are repeated at lines 430 onwards please correct the duplication.

Authors’ response: We have corrected the duplication.

Line 436 – ‘prospective study design’ as above please revise and please check throughout for consistency, clarity and accuracy of reporting.

Authors’ response: We have revised as suggested.

Page 21, line 429-431: “Moreover, reverse causation may to some extent contribute to the observed associations although we tried to minimize the risk for such bias through the study design that simulates a prospective approach.”

REFERENCES
Please check all references for accuracy as per reviewer comments which we agree with – ref #33 appears incomplete, please revise. Refs# 40 and 48 are detailed as ‘invalid citations’. This list is not exhaustive, please check carefully and amend in accordingly.

Authors’ response: We have now updated these named references and gone through the reference list to make sure all references are valid.

Ref #33 updated

Region Stockholms läkemedelskommittés expertgrupp för kvinnosjukdomar och förlossning. Riktlinjer vid premenstruell dysforisk störning, PMDS. 2023-03-13 [cited 25 January 2024]; Available from: <https://janusinfo.se/behandling/expertgruppsutlatanden/kvinnosjukdomarochforlossning/kvinnosjukdomarochforlossning/riktlinjervidpremenstruelldysforiskstorningpmds.5.6081a39c160e9b387319f3.html>.

Ref #40 updated

Brummelte, S. and L.A. Galea, Postpartum depression: Etiology, treatment and consequences for maternal care. Horm Behav, 2016. 77: p. 153-66.

Ref #48 replaced by

Hartlage, S.A., K.E. Arduino, and S. Gehlert, *Premenstrual dysphoric disorder and risk for major depressive disorder: a preliminary study.* J Clin Psychol, 2001. **57**(12): p. 1571-8.

Ref #25 – is listed as ‘in press’ and appears not to have been published. Papers cannot be listed in the reference list until they have been accepted for publication or are publicly available on a preprint archive. Please clarify whether the paper has been accepted for publication in the BMJ, as your bibliography might suggest, and please provide a copy for the editors for reference as well as a letter confirming intent to publish. If you are unable to do the former the information may be cited in the text as a personal communication with the author if the author provides written permission to be named. Alternatively, please provide a different appropriate reference.

Authors’ response: The cited paper has been published January 10th, 2024. We have now updated the citation.
 
Please update your reference formatting in-line with our guidance which can be found here <https://eur01.safelinks.protection.outlook.com/?url=https%3A%2F%2Fjournals.plos.org%2Fplosmedicine%2Fs%2Fsubmission-guidelines%23loc-references&data=05%7C02%7Cqian.yang.1%40ki.se%7C1a5e0612cc4d47843aa908dbffe97dc7%7Cbff7eef1cf4b4f32be3da1dda043c05d%7C0%7C0%7C638385150525725491%7CUnknown%7CTWFpbGZsb3d8eyJWIjoiMC4wLjAwMDAiLCJQIjoiV2luMzIiLCJBTiI6Ik1haWwiLCJXVCI6Mn0%3D%7C3000%7C%7C%7C&sdata=Oy%2FO53Cmp1WQ%2BGaNuzMAhKZHtZnz7%2BDsdCsC3N1mj1M%3D&reserved=0>
In the bibliography, please ensure that you list up to but no more than 6 author names followed by et al.
For all web references please ensure you include an, ‘Accessed [date].’
Journal name abbreviations should be those listed in the National Center for Biotechnology Information (NCBI) databases.

Authors’ response: We have updated the formatting of the “Reference” as required.

SUPPORTING INFORMATION
References - Please ensure that all reference formatting is applied to the supporting information as for the main manuscript. For in-text reference callouts please place citations in square parentheses separate by commas. For example, [1,3,6] or [1-3]. Please check and amend throughout the supporting files.

Authors’ response: We have updated the formatting of the “Reference” in file “Supporting information” as required.

STROBE Checklist – thank you for including the checklist please amend to refer to section and paragraph numbers as opposed to page (or line numbers) as these often change at publication. Please also amend the column header to read ‘Section/paragraph’ or similar.

Authors’ response: We have updated the STROBE checklist included in the file “Supporting information” with section/paragraph of items in the checklist, as suggested.

Tables – as for the main manuscript please separate upper and lower CI bounds with commas as opposed to hyphens as the latter can be confused with reporting of negative values. Please include the statistical test used to determine p values in the footnotes.

Authors’ response: We have separated upper and lower CI bounds with commas as opposed to hyphens as required and added statistical test in the footnotes.

SOCIAL MEDIA
To help us extend the reach of your research, please detail any X (formerly Twitter) handles you wish to be included when we tweet this paper (including your own, your coauthors’, your institution, funder, or lab) in the manuscript submission form when you re-submit the manuscript.

Authors’ response: Thank you for your effort to spread scientific findings through social media. We have included Twitter accounts that we wish to be included in the manuscript submission form.

COMMENTS FROM THE REVIEWERS:

Reviewer #1: Thanks authors for their great effort to improve the manuscript. I am satisfied with the response and revision. No further issues needing attention.

Authors’ response: We thank the reviewer for the acknowledgement of our work on the first revision.

Reviewer #3: The authors have taken a substantial revision of the manuscript which I believe it now meets the standard of PLoS Medicine for publication. Please address the following minor comments before publication.

Authors’ response: Thank you for your positive remarks. We have addressed the following comments as below and hope you find them satisfactory.

1. There are 3 references that are not shown correctly in the bibliography list. Please doublecheck to correct it.

Authors’ response: Thank you for pointing them out. We have updated and/or added details to these references. Please refer to authors’ response to editors’ comment on reference on page 5 in this letter.

2. regarding PND-->PMDs - Line 241 of the Revision 1 version of manuscript, Table 4: how to explain the positive results among those WITH history of other psychiatric disorders (new finding no. 4)? Please elaborate on this.

Authors’ response: We share the reviewer’s interest in the potential role of psychiatric disorders in the observed association. However, we believe that such elaboration is better suited in the “Discussion” section. We have thoroughly discussed possible explanations in the “Discussion” as below:

Page 18, line 373-387: “First, both PMDs and PND have shared liability with psychiatric disorders [2,3], which may explain the findings. PMDs could also lead to the development of psychiatric comorbidities [4], which increase the risk of PND. In this scenario, psychiatric disorders are mediators of the studied associations. Indeed, additional adjustment of psychiatric disorders attenuated the associations. However, the bidirectional associations (relative risks) remained robust and even stronger among women without a history of psychiatric disorders, suggesting our findings cannot be entirely explained by psychiatric disorders. On the other hand, although the absolute risk (e.g., probability or incidence rate) of PMDs is higher among women with a psychiatric history, a diagnosis of PND does not translate into an increased risk of PMDs to the same extent as it does for those without a psychiatric history. This is likely because the already heightened risk of PMDs among women with a psychiatric history, particularly among those with a history of depression. However, PMDs or PND have a relatively weaker correlation with other psychiatric disorders compared to depression [2,3]. Among women with a history of other psychiatric disorders, PMDs appeared to confer a higher risk of PND (Table 3) and *vice versa* (Table 5).”

Reference

1.	Ernster, V.L., *Nested case-control studies.* Prev Med, 1994. **23**(5): p. 587-90.

2.	Pilver, C.E., D.J. Libby, and R.A. Hoff, *Premenstrual dysphoric disorder as a correlate of suicidal ideation, plans, and attempts among a nationally representative sample.* Soc Psychiatry Psychiatr Epidemiol, 2013. **48**(3): p. 437-46.

3.	Beck, C.T., *Predictors of postpartum depression: an update.* Nurs Res, 2001. **50**(5): p. 275-85.

4.	Hartlage, S.A., K.E. Arduino, and S. Gehlert, *Premenstrual dysphoric disorder and risk for major depressive disorder: a preliminary study.* J Clin Psychol, 2001. **57**(12): p. 1571-8.
